# Breaking Down Barriers to a Suicide Prevention Helpline: Web-Based Randomized Controlled Trial

**DOI:** 10.2196/56396

**Published:** 2024-09-05

**Authors:** Margot C A Van der Burgt, Saskia Mérelle, Willem-Paul Brinkman, Aartjan T F Beekman, Renske Gilissen

**Affiliations:** 1 Department of Research, 113 Suicide Prevention, Paasheuvelweg 25, Amsterdam, 1105 BP, The Netherlands, 31 203113883; 2 Department of Psychiatry, Amsterdam University Medical Center, Amsterdam, The Netherlands; 3 Department of Intelligent Systems, Delft University of Technology, Delft, The Netherlands

**Keywords:** barrier reduction intervention, suicidal ideation, self-help, suicide prevention helpline, randomized controlled trial, help-seeking, suicide, RCT, self-test, effectiveness, prevention, middle-aged, behavioral, attitudinal, website visitors, website visitor, website, men, suicide prevention

## Abstract

**Background:**

Every month, around 3800 people complete an anonymous self-test for suicidal thoughts on the website of the Dutch suicide prevention helpline. Although 70% score high on the severity of suicidal thoughts, <10% navigate to the web page about contacting the helpline.

**Objective:**

This study aimed to test the effectiveness of a brief barrier reduction intervention (BRI) in motivating people with severe suicidal thoughts to contact the suicide prevention helpline, specifically in high-risk groups such as men and middle-aged people.

**Methods:**

We conducted a fully automated, web-based, randomized controlled trial. Respondents with severe suicidal thoughts and little motivation to contact the helpline were randomly allocated either to a brief BRI, in which they received a short, tailored message based on their self-reported barrier to the helpline (n=610), or a general advisory text (care as usual as the control group: n=612). Effectiveness was evaluated using both behavioral and attitudinal measurements. The primary outcome measure was the use of a direct link to contact the helpline after completing the intervention or control condition. Secondary outcomes were the self-reported likelihood of contacting the helpline and satisfaction with the received self-test.

**Results:**

In total, 2124 website visitors completed the Suicidal Ideation Attributes Scale and the demographic questions in the entry screening questionnaire. Among them, 1222 were randomized into the intervention or control group. Eventually, 772 respondents completed the randomized controlled trial (intervention group: n=369; control group: n=403). The most selected barrier in both groups was “I don’t think that my problems are serious enough.” At the end of the trial, 33.1% (n=122) of the respondents in the intervention group used the direct link to the helpline. This was not significantly different from the respondents in the control group (144/403, 35.7%; odds ratio 0.87, 95% CI 0.64‐1.18, *P*=.38). However, the respondents who received the BRI did score higher on their self-reported likelihood of contacting the helpline at a later point in time (B=0.22, 95% CI 0.12‐0.32, *P***≤**.001) and on satisfaction with the self-test (B=0.27, 95% CI 0.01‐0.53, *P*=.04). For male and middle-aged respondents specifically, the results were comparable to that of the whole group.

**Conclusions:**

This trial was the first time the helpline was able to connect with high-risk website visitors who were hesitant to contact the helpline. Although the BRI could not ensure that those respondents immediately used the direct link to the helpline at the end of the trial, it is encouraging that respondents indicated that they were more likely to contact the helpline at a later point in time. In addition, this low-cost intervention provided greater insight into the perceived barriers to service. Follow-up research should be focused on identifying the added value of other components (eg, video or photo material) in the BRI and increasing its effectiveness, especially for men and middle-aged people.

## Introduction

Due to its accessibility and anonymity, the internet is often the first place individuals turn to when seeking information on delicate subjects [1,2]. Each month, around 3800 people complete an anonymous self-test for suicidal thoughts on the website of the national suicide prevention helpline in the Netherlands—113 Suicide Prevention [3]. The organization offers 24/7 anonymous phone and chat support, a web-based self-help course, self-assessment tests, as well as brief web-based counseling and therapy. Since its foundation in 2009, brand awareness and service users have increased annually, with more than 151,000 chat and phone call conversations and almost 1.4 million website visits in 2022 [4]. Previous studies on the helpline provided an understanding of its visitors’ profile, with the majority of helpline users being female and younger than 35 years old [5-7].

The self-test for suicidal thoughts consists of the Suicidal Ideation Attributes Scale (SIDAS) and informs the test-taker about the severity of their suicidal ideation by measuring the frequency and controllability of suicidal thoughts, the closeness to an attempt, and the distress and interference with everyday activities [8]. Even though the majority (70%) of test-takers score higher than the threshold for severe suicidal ideation (SIDAS≥21), very few of them (less than 10%) continue to the web page about contacting the helpline by phone or chat. Due to the anonymous nature of the helpline’s services, it is not possible to determine to what extent test-takers follow the advice about contacting the helpline. However, while men make up around 40% of the self-test users, only around 20% of the helpline’s chat users are male [6,7,9].

It is disheartening that so few test-takers go on to contact the helpline by phone or chat, especially given the seriousness of their suicidal thoughts. However, we do know from the literature that a large proportion of individuals with suicidal ideation struggle with seeking adequate help, especially in low-income countries [10,11]. Some known barriers to care are structural factors like time and finances; the lack of perceived need for services; a preference for self-management; fear of hospitalization; and stigmatizing attitudes toward suicide, mental health problems, and seeking professional treatment [10,12]. People may also not receive the care they need because there are not enough services available, they cannot afford the expense of care, or they believe the available services do not meet their needs [13,14]. The study of suicide is complex. Suicidal behavior occurs among vulnerable individuals in the context of a range of different mental illnesses, and social stresses and can be influenced by attitudes toward help-seeking and cultural norms [15]. It is crucial to better understand how to guide high-risk individuals toward professional help. Despite global progress that resulted in a 36% decrease in the age-standardized suicide rate between 2000 and 2019, and no significant increase during the COVID-19 pandemic, suicide remains among the leading causes of death worldwide, with an estimated 703,000 lives lost to suicide in 2019 [16,17]. Globally, over half (58%) of all suicides occurred before the age of 50 years, and the majority of them took place in low- and middle-income countries (77%). Men are at higher risk than women, with a 2.3 higher age-standardised suicide rate than women [17].

This study is focused on suicide prevention in a web-based environment. In this setting, persuasive eHealth technologies can be used to help and motivate people to reach out for help. These persuasive systems can be defined as “computerized software or information systems designed to reinforce, change or shape attitudes or behaviors or both without using coercion or deception” [18]. Little research has been done on stimulating help-seeking behavior among anonymous and high-risk internet users. Therefore, motivating reluctant high-risk individuals in a web-based environment toward professional help is still relatively uncharted territory. With this study, we intended to test whether it is possible to increase service use among individuals with severe suicidal ideation by providing more tailored information. Our study is inspired by the work of Jaroszewski et al [19], in which they evaluated a brief, automated barrier reduction intervention (BRI) designed to increase the use of crisis service referrals provided within the mental health app Koko. To the best of our knowledge, this study is the first automatic, web-based, randomized controlled trial (RCT) among people with severe suicidal ideation that aimed to reduce barriers to a suicide prevention helpline. The aim of our study is 2-fold: (1) to measure the effectiveness of a brief BRI provided in the self-test motivating people with severe suicidal thoughts to contact the Dutch suicide prevention helpline and (2) to specifically evaluate the effectiveness of the BRI in increasing helpline use by high-risk groups for suicide such as men and middle-aged people (40‐70 years) [20].

## Methods

### Study Design

**Figure 1.**
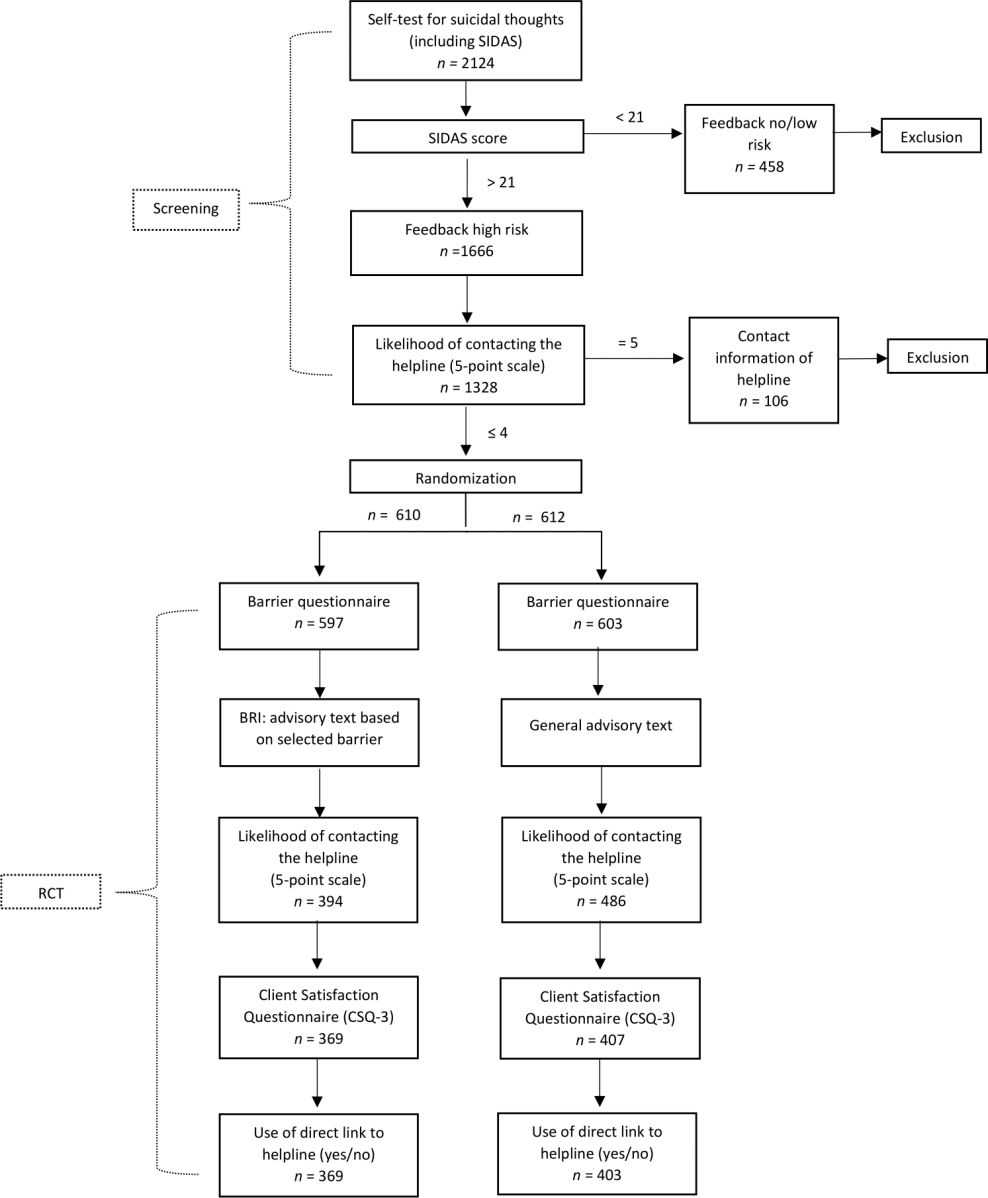
Flowchart. BRI: barrier reduction intervention; CSQ: Client Satisfaction Questionnaire; RCT: randomized controlled trial. SIDAS: Suicidal Ideation Attributes Scale.

This study was designed as an automated, web-based, 2-arm RCT. Respondents with severe suicidal thoughts and little to no interest in contacting the helpline by phone or chat were randomly assigned to either a brief BRI or received a general advisory text (care as usual). It was intended that the intervention could be finished in less than 10 minutes to minimize the burden on our high-risk and sensitive study population. More detailed information about the study’s methodology can be found in the study protocol [21]. Figure 1 displays the study’s flowchart.

### Participants

Participants were recruited between October 7 and December 5, 2022. Anonymous visitors of the self-test for suicidal thoughts on the website of the Dutch national suicide prevention helpline were asked if they wanted to help improve the current self-test by contributing to this study. Visitors of the website could select “Yes, I will participate in the study” or “No, I just want to fill in the self-test.” People who were willing to contribute to our study were redirected to an information page and were asked for their informed consent. Those who were not willing to participate were guided to the already existing self-test on the website.

### Exclusion Criteria

Participants were excluded from the study if: they were younger than 16 years old, scored below the cutoff point for severe suicidal thoughts (SIDAS score <21), or scored above the cutoff point for severe suicidal thoughts (SIDAS score≥21) and reported being likely to contact the suicide prevention helpline. They were directly transferred to the contact details of the helpline. Respondents who did not meet the requirements for inclusion were redirected to a web page thanking them for their time and encouraging them to contact the helpline in case of distress.

### Assessment of Barriers

Both respondents in the intervention and control condition were asked the following question: “Could you indicate why you might not want to talk to one of our counselors at the moment?” Respondents could choose one of the following options: (1) “I don’t think that 113 can help me”; (2) “I’m scared to talk about my feelings”; (3) “I don’t think that my problems are serious enough”; (4) “I’m scared that people will find out”; (5) “I would rather solve it myself”; and the remaining option (6) “I have other reasons.” Answer options were based on a pilot study, more information about the pilot study can be found in the study protocol [21].

### Control Group: Care as Usual

**Figure 2.**
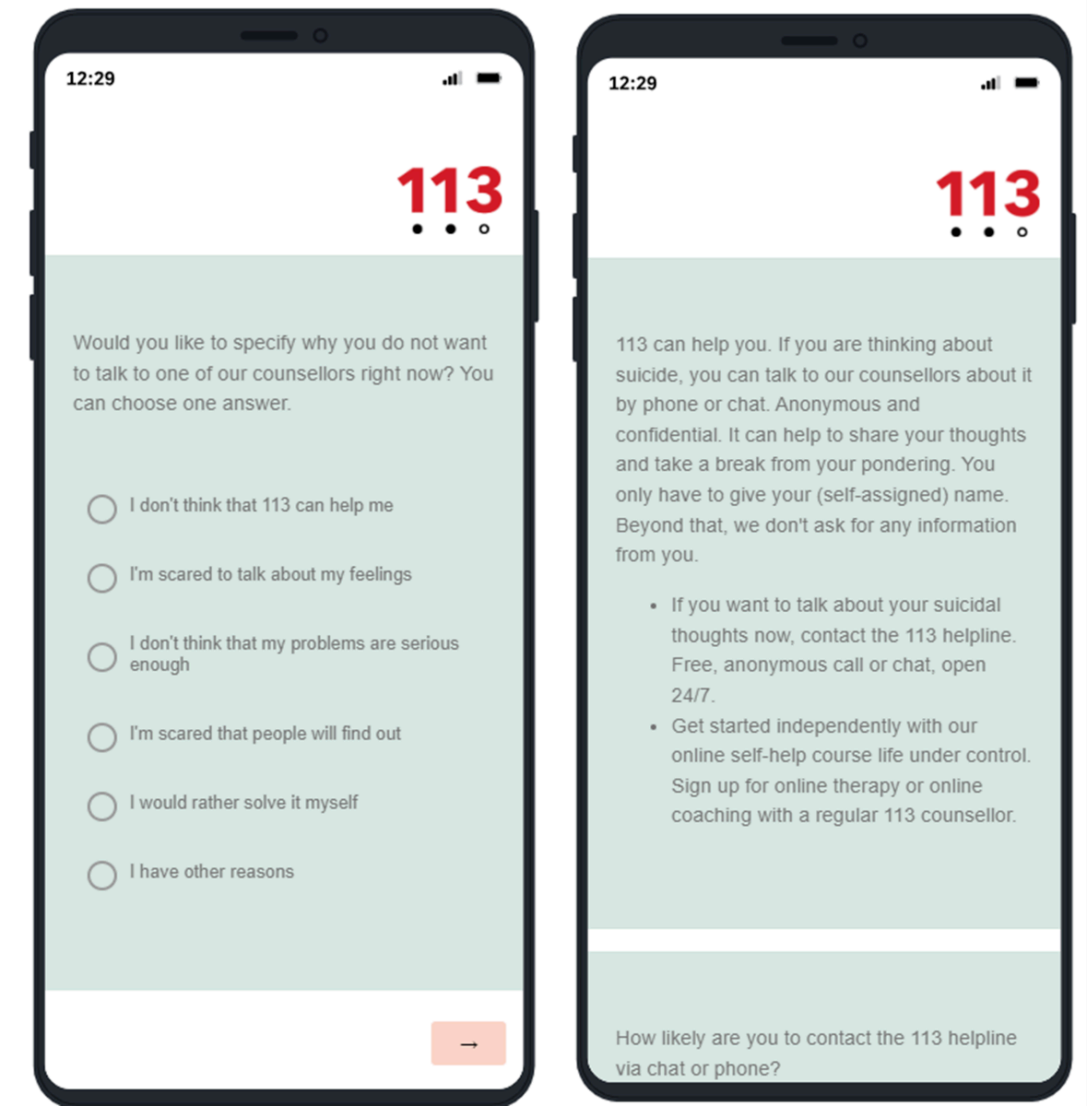
Translated screenshot of the barrier questionnaire (left) and plain advisory text; care as usual (right) received by the control group.

After the barrier question, the control group received a general advisory text. The text was similar to the advisory text individuals receive when they fill in the current self-test on the website of the helpline. Figure 2 shows a translated screenshot.

### Intervention Group: BRI

**Figure 3.**
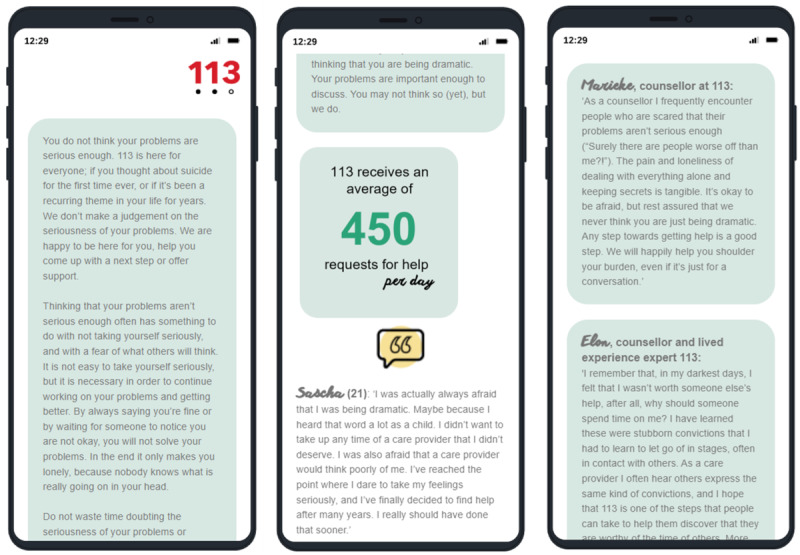
Translated screenshot of the barrier reduction intervention when selecting the barrier “I don’t think my problems are serious enough for 113.”

Respondents in the intervention group received tailored information based on the respondents’ self-reported barrier to contacting the helpline. The tailored information aimed to address common concerns and misconceptions about the helpline. The tailored advisory text for each barrier was structured as follows: (1) a friendly and informal overall advice in the tone of voice of the helpline; (2) an anonymous quote from a help-seeker about his or her experience with help-seeking; and (3) 2 quotes from counselors with or without lived experience. To educate people that they are certainly not the only ones experiencing suicidal thoughts, each advisory text contains a figure showing the text “113 receives an average of 450 requests for help per day.” As per the helpline’s communication policy, each advisory text emphasizes the importance of talking about suicidal thoughts and taking the first step toward help. Figure 3 shows a translated screenshot of the BRI when selecting the barrier “I don’t think that my problems are serious enough for 113.”

### Measurements

#### Screening and Demographic Variables

This study focused on individuals with severe suicidal ideation. This was measured by the SIDAS. This questionnaire consists of 5 items on a 10-point scale measuring the frequency and controllability of suicidal thoughts, the closeness to a suicide attempt, and the distress and interference with daily activities [8]. In terms of demographic data, respondents’ self-reported gender, age group, and treatment status for mental health problems (yes, no, on a waiting list) were collected. One’s likelihood of contacting the helpline before randomization was measured by the question “How likely are you to contact 113’s helpline via chat or phone?” Answering options ranged from “not likely” to “very likely” on a 5-point scale.

#### Primary Outcome Measure: Contacting the Helpline

The use of a direct link to the helpline (yes/no) after completing the intervention or the control condition was the primary outcome measure in this trial. At the end of the RCT, respondents were given a choice between 2 buttons: “exit” or “helpline.”

#### Secondary Outcome Measure: Self-Reported Likelihood of Contacting the Helpline

Due to the helpline’s anonymous nature, it is not possible to determine if those who did not use the link directly after the intervention or control condition, did contact the helpline at a later moment in time. For that reason, a continuous outcome variable was used, that is, the respondent’s self-reported likelihood of contacting the helpline. This was measured with the same likelihood question as in the entry screening questionnaire.

#### Tertiary Outcome Measure: Satisfaction With the Self-Test

Respondents’ satisfaction with the self-test, control and intervention condition, was measured using the Dutch Client Satisfaction Questionnaire (CSQ-3) [22].

### Sample Size

The study protocol specified a sample size of 775 participants. We estimated that approximately 10% of participants would drop out during the intervention. We, therefore, expected that we would need to include at least 853 participants. Furthermore, we anticipated that approximately 30% of test-takers would score below the cutoff point for high risk of suicidal behavior and that 20% of test-takers would indicate a high probability of contacting the helpline in the entry screening questionnaire. Factoring in these projections, we determined that a recruitment goal of at least 1706 respondents was necessary [21].

### Missing Data

Although all questions in the RCT were mandatory to fill in, a total of 450 persons (36.8%) who were randomized did not complete their participation and closed the web page prematurely. Because demographic data were surveyed during the screening phase, before randomization, there were no missing data on gender, age group, being in treatment or not, and severity of suicidal thoughts (SIDAS score).

### Statistical Analyses

The statistical analyses were conducted using R (version 4.1.1; R Foundation for Statistical Computing) and IBM SPSS Statistics (version 25.0). The first step of the data analysis was focused on verifying the randomization process and inspecting dropouts. To determine whether the control group and the intervention group, as well as the completers and noncompleters, were comparable on baseline factors (gender, age, SIDAS score, and in treatment or not) 2-tailed independent *t* tests and *χ*^*2*^ tests were used. To test the hypothesis that respondents who received the brief BRI were more likely to use the direct link to contact the suicide prevention helpline at the end of the trial than respondents in the control condition, *χ*^*2*^ analysis as well as multiple logistic regression analyses were used. For the secondary and tertiary outcome measures, multiple linear regression analyses were conducted.

### Sensitivity Testing

As a sensitivity test, the outcome measures were also analyzed using intention-to-treat analysis. For this analysis, missing values were present in the following variables: barrier to the helpline (22/1222, 1.8%); likelihood of contacting the helpline after the intervention (342/1222, 28%); the CSQ-3 items CSQ1 (421/1222, 34.5%), CSQ2 (428/1222, 35%), and CSQ3 (437/1222, 35.8%); and the main outcome measure (390/1222, 31.9%). All missing values were imputed using the R package MICE [23], generating 10 independent data sets based on a maximum of 10 iterations each. To allow maximum imputation accuracy, we applied the classification and regression trees (cart) method for all variables and did not exclude any variables from the model a priori. Having generated 10 imputed data sets, missing values in the original data set were replaced by the most frequent imputed value for factors, and by the average imputed value for numeric variables.

### Ethical Considerations

This study was reviewed and approved by the Medical Ethics Committee of the Vrije Universiteit Medical Center (registration number: 2021.0443) and is registered at ClinicalTrials.gov (NCT05458830). The study was not subject to the Research Involving Human Subjects Act (*Wet medisch-wetenschappelijk onderzoek met mensen*), as participants were not subject to procedures and were not required to follow rules of behavior. Every participant was directed to a web-based information letter and consent form. After giving consent to participate in the study and stating to be 16 years or older, participants were transferred to the web-based trial. To ensure strict anonymity, no identifying information or IP addresses were gathered.

## Results

### Study Sample

In just under 3 months, 1222 individuals were randomly assigned to the self-test including a brief BRI (n=610) or to the self-test without BRI (care as usual: n=612). Ultimately, 63.2% completed the RCT and answered all questions; 369 in the intervention group and 403 in the control group. The completers and noncompleters were comparable regarding gender, being in treatment for psychological problems, and SIDAS score. The chi-square test revealed a significant difference in the distribution of age groups (*χ*^*2*^_6_=12.65, *P*=.049). Table 1 displays the respondents’ characteristics of those who completed the intervention. The intervention and control groups were comparable regarding age group, being in treatment for psychological problems, and SIDAS score but not on gender (*χ*^*2*^_2_=6.96, *P*=.031), with relatively more men (34.5% vs 28.5%) and fewer people who indicated having a gender other than male or female (3% vs 6.2%) in the control group than in the intervention group. The most selected barrier in both groups was “I don’t think that my problems are serious enough for 113” (Table 2). A Pearson *χ*^*2*^ test was conducted to assess the distribution of the barrier categories between the 2 groups. The test revealed no significant difference (*χ*^*2*^_5_=6.92, *P*=.23). There also appears to be no difference in the perceived barrier toward the helpline between men and women (*χ*^*2*^_5_=7.23, *P*=.20; Table S1 in Multimedia Appendix 1).

**Table 1.** Respondents’ characteristics.

Characteristics		Intervention group (n=369)	Control group (n=403)
**Gender, n (%)**			
	Male	105 (28.5)	139 (34.5)
	Female	241 (65.3)	252 (62.5)
	Other	23 (6.2)	12 (3.0)
**Age group (years), n (%)**			
	16‐24	178 (48.2)	196 (48.6)
	25‐29	41 (11.1)	44 (10.9)
	30‐39	70 (19.0)	55 (13.6)
	40‐49	35 (9.5)	38 (9.4)
	50‐59	30 (8.1)	41 (10.2)
	60‐69	10 (2.7)	18 (4.5)
	≥70	5 (1.4)	11 (2.7)
**Being in treatment, n (%)**			
	Yes	146 (39.6)	176 (43.7)
	No	170 (46.1)	188 (46.7)
	On waiting list	53 (14.4)	39 (9.7)
**SIDAS score**			
	Mean (SD)	32.33 (6.99)	33.19 (7.21)

**Table 2.** Perceived barriers to the helpline per group.

	Intervention group (n=369), n (%)	Control group (n=403)^a^, n (%)
I don’t think that contacting 113 can help me	72 (19.5)	87 (21.6)
I’m scared to talk about my feelings	67 (18.2)	79 (19.6)
I don’t think that my problems are serious enough	115 (31.2)	94 (23.3)
I’m scared that people will find out	41 (11.1)	44 (10.9)
I would rather solve it myself	42 (11.4)	53 (13.2)
I have other reasons	32 (8.7)	46 (11.4)

a No significant difference between the 2 groups; *χ*2_5_=6.92, *P*=.23.

### Main Outcome Measure: The Use of a Direct Link to the Helpline

After completing the intervention, most respondents opted to “exit” the web page instead of using the direct link to the helpline. There was no significant (*χ*^*2*^_1_=.61, *P*=.44) difference between the control group and the intervention group, with 35.7% (n=144) of respondents in the control group using the direct link compared with 33.1% (n=122) in the intervention group. Furthermore, logistic regression analysis (Table 3) showed no differences in gender, SIDAS score, or treatment status but did show that age was a predictor for using the direct link to the helpline (odds ratio [OR] 0.88, 95% CI 0.80‐0.96), with on average, lower odds for the older age groups (Figure S1 in Multimedia Appendix 1). One’s score on the first self-reported question regarding the likelihood of contacting the helpline in the entry screening questionnaire also appears to be of influence (OR 1.23, 95% CI 1.05‐1.43).

**Table 3.** Logistic regression analysis: using the direct link to the helpline (772 complete cases).^a^

		ln(OR)^b^	SE	OR (95% CI)	*P* value
Constant		−1.02	0.44	0.36	.02
**Group (ref: control)**					
	Intervention group	−0.14	0.16	0.87 (0.64‐1.18)	.38
Age group		−0.13	0.05	0.88 (0.80‐0.96)	.01
**Gender (ref: female)**					
	Male	0.08	0.17	1.08 (0.77‐1.52)	.66
	Other	0.19	0.37	1.21 (0.59‐2.48)	.60
SIDAS^c^ score		0.01	0.01	1.01 (0.99‐1.03)	.52
**Treatment status (ref: in treatment)**					
	Not in treatment	0.15	0.17	1.16 (0.83‐1.63)	.37
	On waiting list	−0.04	0.26	0.96 (0.58‐1.59)	.89
Likelihood contact pre-intervention		0.20	0.08	1.23 (1.05‐1.43)	.01

a Nagelkerke *R*2=.031.

b OR: odds ratio.

c SIDAS: Suicidal Ideation Attributes Scale.

### Self-Reported Likelihood of Contacting the Helpline

In the entry screening questionnaire, both the intervention group (mean 2.41, SD 0.96) and control group (mean 2.40, SD 1.00) scored similarly on the self-reported likelihood of contacting the helpline (*t*_770_=.13, *P*=.89). This scale ranges from 1 to 4, as the people who scored “very likely” in the screening phase were not included in the RCT. After the trial, the intervention group (mean 2.78, SD 1.06) scored significantly higher on the 5-point scale than the control group (mean 2.55, SD 1.09*; t*_770_=2.96, *P*=.003). Additionally, the results of the multiple linear regression analysis (Table 4) indicate that an individual’s baseline score on the likelihood of contacting the helpline was a significant contributing factor.

**Table 4.** Regression analysis: self-reported likelihood of contacting the helpline (772 complete cases).^a^

		B	SE	β	95% CI	*P* value
Constant		1.42	0.14	—^b^	1.14 to 1.69	<.001
**Group (ref: control)**						
	Intervention group	0.22	0.05	0.10	0.12 to 0.32	<.001
Age group		−0.03	0.02	−0.05	−0.06 to 0.00	.05
**Gender (ref: female)**						
	Male	−0.07	0.06	−0.03	−0.18 to 0.04	.19
	Other	−0.14	0.12	−0.03	−0.38 to 0.10	.25
SIDAS^c^ score		0.00	0.00	0.01	0.00 to 0.01	.57
**Treatment status (ref: in treatment)**						
	Not in treatment	−0.05	0.06	−0.03	−0.16 to 0.05	.32
	On waiting list	00.00	0.08	0.00	−0.16 to 0.17	.96
Likelihood contact pre-intervention		0.84	0.03	0.76	0.79 to 0.89	<.001

a Adjusted *R*2=0.598.

b Not applicable.

c SIDAS: Suicidal Ideation Attributes Scale.

### Satisfaction With the Self-Test

The respondents in the intervention group scored slightly higher on satisfaction with the self-test (mean 8.55, SD 1.94) than the control group (mean 8.21, SD 1.98; *t*_770_=2.36, *P*=.02). Furthermore, multiple linear regression analysis shows that, in general, the older age groups scored lower on the CSQ scale than the younger age groups. Men (mean 7.93, SD 2.17) were less satisfied with the self-test in the control and intervention conditions than women (mean 8.60, SD 1.79). The results of this regression analysis can be found in Table S5 in Multimedia Appendix 1.

### Evaluating the Effectiveness of the BRI for Men and Those of Middle Age

One-third of the men in the intervention group (35/105, 33%) used the direct link to the helpline, and this is comparable to those in the control group (49/139, 35%; *χ*^*2*^_1_=0.098, *P*=.76). This result did not change after controlling for the different confounders. When we look at the attitudinal measure (Table 5), we see that the intervention group has a positive influence (B=0.20, SE=0.10, *P*=.04) on the self-reported likelihood of contacting the helpline.

**Table 5.** Regression analyses: self-reported likelihood of contacting the helpline for men (244 complete cases).^a^

		B	SE	β	95% CI	*P* value
Constant		0.09	0.26	—^b^	−0.42 to 0.59	.74
**Group (ref: control)**						
	Intervention group	0.20	0.10	0.09	0.01 to 0.39	.04
Age group		−0.02	0.03	−0.04	−0.07 to 0.03	.42
SIDAS^c^ score		0.01	0.01	0.05	−0.01 to 0.02	.23
**Treatment status (ref: in treatment)**						
	Not in treatment	0.04	0.11	0.02	−0.17 to 0.25	.72
	On waiting list	0.05	0.19	0.01	−0.31 to 0.42	.78
Likelihood contact pre-intervention		0.83	0.05	0.75	0.74 to 0.93	<.001

a Adjusted *R*2=0.55.

b Not applicable.

c SIDAS: Suicidal Ideation Attributes Scale.

Among the 172 middle-aged respondents (40‐70 years), 32% (n=24) of the intervention group and 27% (n=26) in the control group used the direct link to the helpline. This difference was too small to be significant (*χ*^*2*^_1_=0.55, *P*=.46) and did not change after controlling for the different confounders. On the attitudinal measure, the intervention group had a mean score of 2.63 (SD 0.91) regarding the likelihood of contacting the helpline, while the control group showed a slightly lower mean score of 2.48 (SD 0.98). When controlled for the various confounders, this difference was significant (Table 6).

**Table 6.** Regression analyses: self-reported likelihood of contacting the helpline for middle-aged respondents (172 complete cases).^a^

		B	SE	β	95% CI	*P* value
Constant		−0.06	0.29	—^b^	−0.64 to 0.51	.83
**Group (ref: control)**						
	Intervention group	0.22	0.10	0.12	0.02 to 0.43	.03
**Gender (ref: female)**						
	Male	−0.01	0.11	−0.01	−0.22 to 0.19	.89
	Other	0.22	0.38	0.03	−0.54 to 0.98	.56
SIDAS^c^ score		0.02	0.01	0.12	0.00 to 0.03	.03
**Treatment status (ref: in treatment)**						
	Not in treatment	−0.09	0.11	−0.05	−0.32 to 0.13	.41
	On waiting list	0.09	0.17	0.03	−0.24 to 0.43	.59
Likelihood contact pre-intervention		0.72	0.05	0.71	0.61 to 0.83	<.001

a Adjusted *R*2=0.53.

b Not applicable.

c SIDAS: Suicidal Ideation Attributes Scale.

### Sensitivity Test

As a sensitivity test, the outcome measures were also analyzed using intention-to-treat analysis. When using the imputed data set (n=1222) for our regression models, the outcomes were consistently confirmed. The only difference can be found in the regression analysis on self-reported likelihood of contacting the helpline for men. When using the imputed data, the significant difference between the intervention and control groups disappeared. The summaries of the regression models can be found in Tables S3-S9 in Multimedia Appendix 1

## Discussion

### Principal Findings

The aim of our study was 2-fold: (1) to measure the effectiveness of a brief BRI provided in the self-test motivating people with severe suicidal thoughts to contact the Dutch suicide prevention helpline and (2) to specifically evaluate the effectiveness of the BRI in increasing service use by high-risk groups for suicide such as men and middle-aged people.

This study was the first occasion in which respondents were actively recruited among the anonymous website visitors of the Dutch suicide prevention helpline. Recruiting the respondents went faster than expected; in just under 3 months, we had reached the targeted number of respondents. Respondents’ characteristics were in line with expectations based on previous research on the helpline. They were mainly female, young, and having severe suicidal thoughts [5-7]. Almost half of the respondents (358/772, 46.4%) were not receiving treatment from mental health services at the time. This percentage was higher among the male (148/244, 60.7%) than the female respondents (192/493, 38.9%). At the time of the study, 11.9% (n=92) of the respondents were on a waiting list for mental health services (men: 21/244, 8.6%; women: 68/493, 13.8%). The higher percentage of men not receiving treatment from mental health services for suicidality is in line with previous research [10,24,25]. These gender differences in help-seeking behavior are likely to be a factor behind the higher number of suicides among men compared with women [26,27]. During the pilot study in 2021, 7252 people filled in the self-test. The majority of them (n=5200, 72%) scored higher than the cutoff point for severe suicidal thoughts, and the mean SIDAS score was 27 on a scale ranging from 0 to 50 [21]. In this study, 2124 website visitors completed the SIDAS and the demographic questions in the screening phase. More than three-quarters (78%) scored higher than the cutoff point for severe suicidal thoughts (SIDAS≥21). The mean SIDAS score in the screening phase was 28.4. Among the eventual study sample, the mean SIDAS score was 33, as the BRI was intended for the high-risk group.

The most selected barriers to the helpline in both groups were “I don’t think that my problems are serious enough,” “I don’t think that 113 can help me,” and “I’m scared to talk about my feelings.” These barriers align with known barriers to seeking professional help in people with suicidal ideation, such as a low perceived need for support, a strong inclination toward self-reliance and stigmatizing attitudes toward suicide, mental health issues, and professional treatment [10]. The results indicated no difference in the use of a direct link to the helpline immediately at the end of the few-minute–long trial. However, the results do show an impact on one’s self-reported likelihood of contacting the helpline. In addition, respondents who received the BRI were more positive about the self-test than those in the control condition. When looking at the high-risk groups men and middle-aged people specifically, the results were comparable to the whole group of respondents. Brief eHealth interventions tend to struggle with achieving significant effects on behavioral outcomes [28,29]. In their review of methods for human-centered eHealth development, Kip et al [30] describe many varieties of methods and products that can be used throughout the development process to prioritize user perspectives to enhance the effectiveness and usability of eHealth solutions.

### Limitations

Although barriers to help-seeking have been studied in traditional mental health care, persuading reluctant individuals with severe suicidal ideation in a web-based environment toward professional help is still a largely unexplored area. Therefore, this study, with its randomized controlled design, large sample sizes, and hard-to-reach and at-risk study population, brings a valuable contribution to the suicide prevention literature. However, this study has some limitations that should be taken into consideration when interpreting its results. First and most importantly, the BRI in this study was only text-based. For future research, it would be valuable to determine the effects of different types of components (eg, video material) in a similar BRI. Second, due to the anonymity of the suicide prevention helpline, it was not possible to include a follow-up measurement. It is therefore not feasible to determine if those who did not use the link to the helpline after the intervention did contact the helpline at a later moment in time. Because of this, the self-reported likelihood of contacting the helpline has also been measured as well as the use of the direct link. Third, there were relatively high dropout rates during the RCT (36.8%). This is not entirely unexpected for a short, anonymous, web-based survey but still noteworthy. In order to assess the robustness of the results, analyses were carried out on the complete cases (per protocol) as well as on an imputed data set (intention-to-treat). Finally, we recognize the importance of involving people with lived and living experiences of suicidality in research. Although the content and barriers in the RCT were based on a qualitative pilot study among the self-test users, it would be desirable to involve people with lived experience throughout the research process [21]. To give researchers the confidence and willingness to involve the research population, Orygen developed guidelines for safely and effectively involving young people with lived experience in research [31].

### Practical Implications and Future Research

This short text-based BRI was not effective in inducing direct behavioral change. However, this low-cost and low-effort method to reach those who are highly at risk and are reluctant to contact helpline services gives more insight into the perceived barriers to service. The 5 barriers in the RCT were chosen based on a pilot study among self-test users. These barriers may differ in different cultural and social contexts. For future research, it would be advantageous to join efforts with other helplines to see how much these barriers vary between countries. Furthermore, it would have added value to monitor helpline use for subgroups after the launch of a BRI. In addition, it would also be useful to seek input from help seekers who have had contact with the helpline, explore their experiences during interactions, and identify effective communication strategies. Our follow-up research is focused on using video materials in the BRI.

### Conclusions

The short BRI, built in a self-test for suicidal thoughts, aimed to persuade individuals with severe suicidal ideation to contact the Dutch suicide prevention helpline. Although the BRI could not induce direct behavioral change in our population with severe suicidal thoughts, these few minutes of intervention did manage to increase the self-reported likelihood of contacting the helpline. When tailored to its users, a BRI may have the potential to be a low-cost, highly scalable, and easily implementable method to increase service use for helplines worldwide.

## Supplementary material

10.2196/56396 Multimedia Appendix 1 Supplementary materials regarding perceived barriers, use of the direct link to the helpline, and sensitivity analyses.

10.2196/56396 Checklist 1 CONSORT-eHEALTH checklist (V 1.6.1).

## Abbreviations

BRI: barrier reduction intervention
CSQ: Client Satisfaction Questionnaire
OR: odds ratio
RCT: randomized controlled trial
SIDAS: Suicidal Ideation Attributes Scale
