# Supplementary material for: Breaking Down Barriers to a Suicide Prevention Helpline: Web-Based Randomized Controlled Trial
Source: JMIR Ment Health. 2024 Sep 5;11:e56396. doi: 10.2196/56396 (PMC11391658; doi:10.2196/56396)
Supplement: Multimedia Appendix 1 [file mental-v11-e56396-s001.docx]

# Multimedia Appendix

This is a Multimedia Appendix to a full manuscript:
Breaking down barriers to a suicide prevention helpline: a web-based randomized controlled trial

Margot C.A. van der Burgt^1,2^, Saskia Mérelle^1^, Willem-Paul Brinkman^3^, Aartjan T.F. Beekman^2^, Renske Gilissen^1^

^1^ Department of Research, 113 Suicide Prevention, Amsterdam, the Netherlands;
^2^ Department of Psychiatry, Amsterdam University Medical Center, Amsterdam, the Netherlands;
^3^ Department of Intelligent Systems, Delft University of Technology, Delft, the Netherlands;

Table S1. The perceived barrier to the helpline by gender

|  | Female  (*N* = 493) | Male^a, b^  (*N* = 244) |
| --- | --- | --- |
| I don’t think that contacting 113 can help me | 19.7% | 23.8% |
| I’m scared to talk about my feelings | 18.1% | 20.9% |
| I don’t think that my problems are serious enough | 29.4% | 21.3% |
| I’m scared that people will find out | 11.0% | 9.8% |
| I would rather solve it myself | 12.0% | 14.8% |
| I have other reasons | 9.9% | 9.4% |

^a^ The group whose gender was ‘other’ was too little to break down by selected barrier.

^b^ No significant difference between the two groups; ꭓ2 = 7.23, df = 5, *P* = .20

Figure S1. Use of the direct link to the helpline by age group

Table S2. Regression analysis; satisfaction with the self-test

|  | B | SE | Beta | 95% CI | *P* value |
| --- | --- | --- | --- | --- | --- |
| Constant | 8.64^a^ | 0.37 |  | 7.91 – 9.38 | <.001 |
| **Group (ref: control)** |  |  |  |  |  |
| Intervention group | 0.27 | 0.13 | 0.07 | 0.01 – 0.53 | .044 |
| Age group | -0.20 | 0.04 | -0.17 | -0.28 – -0.12 | <.001 |
| **Gender (ref: female)** |  |  |  |  |  |
| Male | -0.46 | 0.15 | -0.11 | -0.75 – -0.17 | .002 |
| Other | -0.31 | 0.32 | -0.03 | -0.94 – 0.33 | .344 |
| SIDAS score | -0.02 | 0.01 | -0.07 | -0.04 – -0.00 | .049 |
| **Treatment status (ref: in treatment)** |  |  |  |  |  |
| Not in treatment | -0.22 | 0.15 | -0.06 | -0.51 – 0.07 | .130 |
| On waiting list | -0.09 | 0.22 | -0.01 | -0.52 – 0.34 | .691 |
| Likelihood contact pre-intervention | 0.54 | 0.07 | 0.27 | 0.41 – 0.68 | <.001 |
|  |  |  |  |  |  |
| Adjusted R^2^ | 0.13 |  |  |  |  |
| *N* = 772, complete cases |  |  |  |  |  |

^a^CSQ-3 ranges between 3 and 12.

## Sensitivity analyses

As a sensitivity test, the outcome measures were also analyzed using intention-to-treat analysis. Using imputed data, N = 1,222

### Main outcome measure; the use of a direct link to the helpline

Table S3. Logistic Regression Analysis; using the direct link to the helpline

|  | In(OR) | SE | OR (95% CI) | *P* value |
| --- | --- | --- | --- | --- |
| Constant | -1.27 | 0.36 | 0.28 | <.001 |
| Group (ref: control) |  |  |  |  |
| Intervention group | -0.20 | 0.13 | 0.82 (0.63-1.05) | .122 |
| Age group | -0.13 | 0.04 | 0.88 (0.81-0.96) | .004 |
| Gender (ref: female) |  |  |  |  |
| Male | 0.12 | 0.14 | 1.13 (0.85-1.49) | .398 |
| Other | 0.27 | 0.31 | 1.31 (0.71-2.41) | .391 |
| SIDAS score | 0.00 | 0.01 | 1.00 (0.99-1.02) | .726 |
| Treatment status (ref: in treatment) |  |  |  |  |
| Not in treatment | 0.15 | 0.14 | 1.16 (0.88-1.54) | .301 |
| On waiting list | -0.22 | 0.21 | 0.80(0.53-1.22) | .310 |
| Likelihood contact pre-intervention | 0.30 | 0.06 | 1.34(1.18-1.53) | <.001 |
|  |  |  |  |  |
| Nagelkerke R^2^ | .041 |  |  |  |

### Self-reported likelihood of contacting the helpline

Table S4. Regression analysis: self-reported likelihood of contacting the helpline

|  | B | SE | Beta | 95% CI | *P* value |
| --- | --- | --- | --- | --- | --- |
| Constant | 0.54 | 0.11 |  | 0.33-0.75 | <.001 |
| Group (ref: control) |  |  |  |  |  |
| Intervention group | 0.18 | 0.04 | 0.09 | 0.11-0.26 | <.001 |
| Age group | -0.02 | 0.01 | -0.04 | -0.05-0.00 | .044 |
| Gender (ref: female) |  |  |  |  |  |
| Male | -0.10 | 0.04 | -0.05 | -0.18- -0.01 | .023 |
| Other | -0.18 | 0.10 | -0.04 | -0.37-0.01 | .059 |
| SIDAS score | 0.00 | 0.00 | 0.02 | 0.00-0.01 | .354 |
| Treatment status (ref: in treatment) |  |  |  |  |  |
| Not in treatment | -0.02 | 0.04 | -0.01 | -0.10-0.07 | .719 |
| On waiting list | -0.01 | 0.06 | 0.00 | -0.13-0.11 | .870 |
| Likelihood contact pre-intervention | 0.72 | 0.02 | 0.73 | 0.68-0.76 | <.001 |
|  |  |  |  |  |  |
| Adjusted R^2^ | .548 |  |  |  |  |

### Satisfaction with the self-test

Table S5. Regression analysis: satisfaction with the self-test

|  | B | SE | Beta | 95% CI | *P* value |
| --- | --- | --- | --- | --- | --- |
| Constant | 5.71 | 0.25 |  | 5.22-6.20 | <.001 |
| Group (ref: control) |  |  |  |  |  |
| Intervention group | 0.22 | 0.09 | 0.07 | 0.05-0.40 | .014 |
| Age group | -0.16 | 0.03 | -0.15 | -0.22- -0.10 | <.001 |
| Gender (ref: female) |  |  |  |  |  |
| Male | -0.35 | 0.10 | -0.10 | -0.54- -0.16 | <.001 |
| Other | -0.37 | 0.23 | -0.04 | -0.81-0.08 | .104 |
| SIDAS score | -0.02 | 0.01 | -0.08 | -0.03- -0.01 | .005 |
| Treatment status (ref: in treatment) |  |  |  |  |  |
| Not in treatment | -0.23 | 0.10 | -0.07 | -0.42 -0.03 | .022 |
| On waiting list | -0.15 | 0.14 | -0.03 | -0.43-0.13 | .300 |
| Likelihood contact pre-intervention | 0.46 | 0.05 | 0.28 | 0.38-0.55 | <.001 |
|  |  |  |  |  |  |
| Adjusted R^2^ | .128 |  |  |  |  |

Note: CSQ-3 ranges between 0 and 9.

### Evaluating the effectiveness of the BRI for men and those of middle age

Table S6. Logistic Regression Analysis; using the direct link to the helpline for men

|  | In(OR) | SE | OR (95% CI) | *P* value |
| --- | --- | --- | --- | --- |
| Constant | -1.24 | 0.62 | 0.29 | .046 |
| Group (ref: control) |  |  |  |  |
| Intervention group | 0.06 | 0.22 | 1.06 (0.68-1.64) | .797 |
| Age group | -0.14 | 0.07 | 0.87 (0.76-0.99) | .033 |
| SIDAS score | 0.01 | 0.02 | 1.01 (0.98-1.04) | .534 |
| Treatment status (ref: in treatment) |  |  |  |  |
| Not in treatment | 0.03 | 0.25 | 1.03 (0.62-1.69) | .917 |
| On waiting list | -0.86 | 0.44 | 0.42 (0.18-1.01) | .052 |
| Likelihood contact pre-intervention | 0.23 | 0.11 | 1.26 (1.01-1.58) | .038 |
|  |  |  |  |  |
| Nagelkerke R^2^ | .046 |  |  |  |
| *N* = 404 |  |  |  |  |

Table S7. Logistic Regression Analysis; using the direct link to the helpline for middle aged (40-70 years) respondents

|  | In(OR) | SE | OR (95% CI) | *P* value |
| --- | --- | --- | --- | --- |
| Constant | -2.80 | 0.93 | 0.06 | .002 |
| Group (ref: control) |  |  |  |  |
| Intervention group | 0.27 | 0.31 | 1.31 (0.71-2.41) | .389 |
| Gender (ref: female) |  |  |  |  |
| Male | -0.29 | 0.33 | 0.75 (0.39-1.42) | .376 |
| Other | 0.53 | 1.27 | 1.70 (0.14-20.27) | .676 |
| SIDAS score | 0.03 | 0.03 | 1.03 (0.98-1.09) | .194 |
| Treatment status (ref: in treatment) |  |  |  |  |
| Not in treatment | 0.53 | 0.35 | 1.70 (0.86-3.35) | .129 |
| On waiting list | -0.81 | 0.66 | 0.45 (0.12-1.63) | .221 |
| Likelihood contact pre-intervention | 0.26 | 0.16 | 1.29 (0.94-1.77) | .112 |
|  |  |  |  |  |
| Nagelkerke R^2^ | .057 |  |  |  |
| *N* = 251 |  |  |  |  |

Table S8. Regression analyses: self-reported likelihood of contacting the helpline for men

|  | B | SE | Beta | 95% CI | *P* value |
| --- | --- | --- | --- | --- | --- |
| Constant | 0.47 | 0.19 |  | 0.10-0.85 | .014 |
| Group (ref: control) |  |  |  |  |  |
| Intervention group | 0.13 | 0.07 | 0.06 | -0.01-0.26 | .069 |
| Age group | -0.02 | 0.02 | -0.03 | -0.06-0.02 | .427 |
| SIDAS score | 0.00 | 0.01 | 0.02 | -0.01-0.01 | .640 |
| Treatment status (ref: in treatment) |  |  |  |  |  |
| Not in treatment | -0.01 | 0.08 | -0.01 | -0.17-0.14 | .867 |
| On waiting list | 0.05 | 0.12 | 0.02 | -0.19-0.28 | .691 |
| Likelihood contact pre-intervention | 0.71 | 0.04 | 0.71 | 0.64-0.78 | <.001 |
|  |  |  |  |  |  |
| Adjusted R^2^ | 0.506 |  |  |  |  |
| *N* = 404 |  |  |  |  |  |

Table S9. Regression analyses: self-reported likelihood of contacting the helpline for middle-aged respondents

|  | B | SE | Beta | 95% CI | *P* value |
| --- | --- | --- | --- | --- | --- |
| Constant | 0.09 | 0.23 |  | -0.36-0.54 | .693 |
| Group (ref: control) |  |  |  |  |  |
| Intervention group | 0.20 | 0.08 | 0.11 | 0.05-0.36 | .012 |
| Gender (ref: female) |  |  |  |  |  |
| Male | -0.08 | 0.08 | -0.04 | -0.25-0.08 | .324 |
| Other | 0.17 | 0.37 | 0.02 | -0.56-0.89 | .654 |
| SIDAS score | 0.01 | 0.01 | 0.10 | 0.00-0.03 | .021 |
| Treatment status (ref: in treatment) |  |  |  |  |  |
| Not in treatment | -0.01 | 0.09 | -0.01 | -0.19-0.16 | .888 |
| On waiting list | 0.02 | 0.13 | 0.01 | -0.24-0.28 | .877 |
| Likelihood contact pre-intervention | 0.69 | 0.04 | 0.72 | 0.61-0.77 | <.001 |
|  |  |  |  |  |  |
| Adjusted R^2^ | .539 |  |  |  |  |
| *N* = 251 |  |  |  |  |  |
