# Supplementary material for: Breaking Down Barriers to a Suicide Prevention Helpline: Web-Based Randomized Controlled Trial
Source: JMIR Ment Health. 2024 Sep 5;11:e56396. doi: 10.2196/56396 (PMC11391658; doi:10.2196/56396)
Supplement: Checklist 1 [file mental-v11-e56396-s002.pdf]

CONSORT-EHEALTH (V 1.6.1) - Submission/Publication Form

The CONSORT-EHEALTH checklist is intended for authors of randomized trials evaluating web-based and Internet-based applications/interventions, including mobile interventions, electronic games (incl multiplayer games), social media, certain telehealth applications, and other interactive and/or networked electronic applications. Some of the items (e.g. all subitems under item 5 - description of the intervention) may also be applicable for other study designs.

The goal of the CONSORT EHEALTH checklist and guideline is to be
a) a guide for reporting for authors of RCTs,
b) to form a basis for appraisal of an ehealth trial (in terms of validity)

CONSORT-EHEALTH items/subitems are MANDATORY reporting items for studies published in the Journal of Medical Internet Research and other journals / scientific societies endorsing the checklist.

Items numbered 1., 2., 3., 4a., 4b etc are original CONSORT or CONSORT-NPT (non-pharmacologic treatment) items.
Items with Roman numerals (i., ii, iii, iv etc.) are CONSORT-EHEALTH extensions/clarifications.

As the CONSORT-EHEALTH checklist is still considered in a formative stage, we would ask that you also RATE ON A SCALE OF 1-5 how important/useful you feel each item is FOR THE PURPOSE OF THE CHECKLIST and reporting guideline (optional).

Mandatory reporting items are marked with a red *.
[illegible]e textboxes, either copy & paste the relevant sections from your manuscript into this [illegible] - please include any quotes from your manuscript in QUOTATION MARKS, [illegible]nswer directly by providing additional information not in the manuscript, or elaborating [illegible]hy the item was not relevant for this study.

scite_
1459
4
1011
1

[illegible]R ANSWERS WILL BE PUBLISHED AS A SUPPLEMENTARY FILE TO YOUR [illegible]BLICATION IN JMIR AND ARE CONSIDERED PART OF YOUR PUBLICATION (IF ACCEPTED).
Please fill in these questions diligently. Information will not be copyedited, so please use proper spelling and grammar, use correct capitalization, and avoid abbreviations.

DO NOT FORGET TO SAVE AS PDF _AND_ CLICK THE SUBMIT BUTTON SO YOUR ANSWERS ARE IN OUR DATABASE !!!

Citation Suggestion (if you append the pdf as Appendix we suggest to cite this paper in the caption):
Eysenbach G, CONSORT-EHEALTH Group
CONSORT-EHEALTH: Improving and Standardizing Evaluation Reports of Web-based and Mobile Health Interventions
J Med Internet Res 2011;13(4):e126

URL: http://www.jmir.org/2011/4/e126/
doi: 10.2196/jmir.1923
PMID: 22209829

m.vanderburgt@113.nl Ander account

Niet gedeeld

Concept opgeslagen

* Verplichte vraag

Your name *

First Last

Van der Burgt

Primary Affiliation (short), City, Country *

University of Toronto, Toronto, Canada

113 Suicide Prevention

Your e-mail address *

abc@gmail.com

scite_
1459
4
1011
1

[illegible]nderburgt@113.nl

Title of your manuscript *

Provide the (draft) title of your manuscript.

Breaking down barriers to a suicide prevention helpline: a web-based randomized controlled trial

Name of your App/Software/Intervention *

If there is a short and a long/alternate name, write the short name first and add the long name in brackets.

Barrier Reduction Intervention

Evaluated Version (if any)

e.g. "V1", "Release 2017-03-01", "Version 2.0.27913"

Jouw antwoord

Language(s) *

What language is the intervention/app in? If multiple languages are available, separate by comma (e.g. "English, French")

Dutch

URL of your Intervention Website or App

e.g. a direct link to the mobile app on app in appstore (itunes, Google Play), or URL of the [illegible]site. If the intervention is a DVD or hardware, you can also link to an Amazon page.

[illegible]v antwoord

scite_
1459
4
1011
1

URL of an image/screenshot (optional)

Jouw antwoord

Accessibility *

Can an enduser access the intervention presently?

- ◯ access is free and open
- ◯ access only for special usergroups, not open
- ◯ access is open to everyone, but requires payment/subscription/in-app purchases
- ◉ app/intervention no longer accessible
- ◯ Anders:

Primary Medical Indication/Disease/Condition *

e.g. "Stress", "Diabetes", or define the target group in brackets after the condition, e.g. "Autism (Parents of children with)", "Alzheimers (Informal Caregivers of)"

suicidality (severe suicidal thoughts)

Primary Outcomes measured in trial *

comma-separated list of primary outcomes reported in the trial

[illegible]use of a direct link to contact the suicide p

scite_
1459
4
1011
1

[illegible]ondary/other outcomes

[illegible] there any other outcomes the intervention is expected to affect?

"Self-reported likelihood of contacting the helpline and satisfaction with the received self-test".

Recommended "Dose" *

What do the instructions for users say on how often the app should be used?

- Approximately Daily
- Approximately Weekly
- Approximately Monthly
- Approximately Yearly
- "as needed"
- Anders: brief, onetime intervention

Approx. Percentage of Users (starters) still using the app as recommended after 3 months *

- unknown / not evaluated
- 0-10%
- 11-20%
- 21-30%
- 31-40%
- 41-50%
- 51-60%
- 61-70%
- 71%-80%
- 81-90%
- 91-100%
- Anders: not applicable

scite_
1459
4
1011
1

Overall, was the app/intervention effective? *

- ( ) yes: all primary outcomes were significantly better in intervention group vs control
- ( ) partly: SOME primary outcomes were significantly better in intervention group vs control
- (•) no statistically significant difference between control and intervention
- ( ) potentially harmful: control was significantly better than intervention in one or more outcomes
- ( ) inconclusive: more research is needed
- ( ) Anders:

Article Preparation Status/Stage *

At which stage in your article preparation are you currently (at the time you fill in this form)

- ( ) not submitted yet - in early draft status
- ( ) not submitted yet - in late draft status, just before submission
- ( ) submitted to a journal but not reviewed yet
- (•) submitted to a journal and after receiving initial reviewer comments
- ( ) submitted to a journal and accepted, but not published yet
- ( ) published
- ( ) Anders:

scite_
1459
4
1011
1

Journal *

If you already know where you will submit this paper (or if it is already submitted), please provide the journal name (if it is not JMIR, provide the journal name under "other")

- ( ) not submitted yet / unclear where I will submit this
- (x) Journal of Medical Internet Research (JMIR)
- ( ) JMIR mHealth and UHealth
- ( ) JMIR Serious Games
- ( ) JMIR Mental Health
- ( ) JMIR Public Health
- ( ) JMIR Formative Research
- ( ) Other JMIR sister journal
- ( ) Anders:

Is this a full powered effectiveness trial or a pilot/feasibility trial? *

- ( ) Pilot/feasibility
- (x) Fully powered

scite_
1459
4
1011
1

[illegible]nuscript tracking number *

[illegible]s is a JMIR submission, please provide the manuscript tracking number under "other" (The ms tracking number can be found in the submission acknowledgement email, or when you login as author in JMIR. If the paper is already published in JMIR, then the ms tracking number is the four-digit number at the end of the DOI, to be found at the bottom of each published article in JMIR)

- ( ) no ms number (yet) / not (yet) submitted to / published in JMIR
- (x) Anders: 56396

TITLE AND ABSTRACT

1a) TITLE: Identification as a randomized trial in the title

1a) Does your paper address CONSORT item 1a? *

I.e does the title contain the phrase "Randomized Controlled Trial"? (if not, explain the reason under "other")

- (selected) yes
- Anders:

1a-i) Identify the mode of delivery in the title

Identify the mode of delivery. Preferably use “web-based” and/or “mobile” and/or “electronic game” in the title. Avoid ambiguous terms like “online”, “virtual”, “interactive”. Use “Internet-based” only if Intervention includes non-web-based Internet components (e.g. email), use “computer-based” or “electronic” only if offline products are used. Use “virtual” only in the context of “virtual reality” (3-D worlds). Use “online” only in the context of “online support groups”. Complement or substitute product names with broader terms for the class of products (such as “mobile” or “smart phone” instead of “iphone”), especially if the application runs on different platforms.

scite_
1459
4
1011
1

	1	2	3	4	5	
bitem not at all important	○	○	○	○	○	essential

Does your paper address subitem 1a-i? *

Copy and paste relevant sections from manuscript title (include quotes in quotation marks "like this" to indicate direct quotes from your manuscript), or elaborate on this item by providing additional information not in the ms, or briefly explain why the item is not applicable/relevant for your study

"a web-based randomized controlled trial"

1a-ii) Non-web-based components or important co-interventions in title

Mention non-web-based components or important co-interventions in title, if any (e.g., “with telephone support”).

	1	2	3	4	5	
subitem not at all important	○	○	○	○	○	essential

Does your paper address subitem 1a-ii?

Copy and paste relevant sections from manuscript title (include quotes in quotation marks "like this" to indicate direct quotes from your manuscript), or elaborate on this item by providing additional information not in the ms, or briefly explain why the item is not applicable/relevant for your study

Jouw antwoord

1a-iii) Primary condition or target group in the title

Mention primary condition or target group in the title, if any (e.g., “for children with Type I Diabetes”) Example: A Web-based and Mobile Intervention with Telephone Support for Children with Type I Diabetes: Randomized Controlled Trial

	1	2	3	4	5	
subitem not at all important	○	○	○	○	○	essential

scite_
1459
4
1011
1

Does your paper address subitem 1a-iii? *

Copy and paste relevant sections from manuscript title (include quotes in quotation marks "like this" to indicate direct quotes from your manuscript), or elaborate on this item by providing additional information not in the ms, or briefly explain why the item is not applicable/relevant for your study

"Breaking down barriers to a suicide prevention helpline"

1b) ABSTRACT: Structured summary of trial design, methods, results, and conclusions

NPT extension: Description of experimental treatment, comparator, care providers, centers, and blinding status.

1b-i) Key features/functionalities/components of the intervention and comparator in the METHODS section of the ABSTRACT

Mention key features/functionalities/components of the intervention and comparator in the abstract. If possible, also mention theories and principles used for designing the site. Keep in mind the needs of systematic reviewers and indexers by including important synonyms. (Note: Only report in the abstract what the main paper is reporting. If this information is missing from the main body of text, consider adding it)

	1	2	3	4	5	
subitem not at all important	○	○	○	○	○	essential

Does your paper address subitem 1b-i? *

Copy and paste relevant sections from the manuscript abstract (include quotes in quotation marks "like this" to indicate direct quotes from your manuscript), or elaborate on this item by providing additional information not in the ms, or briefly explain why the item is not applicable/relevant for your study

scite_
1459
4
1011
1

[illegible]pondents with severe suicidal thoughts and little motivation to contact the helpline [illegible] randomly allocated either to a brief BRI, in which they received a short, tailored [illegible]sage based on their self-reported barrier to the helpline (N = 610) or a general advisory (care as usual as control group, N = 612)".

1b-ii) Level of human involvement in the METHODS section of the ABSTRACT

Clarify the level of human involvement in the abstract, e.g., use phrases like “fully automated” vs. “therapist/nurse/care provider/physician-assisted” (mention number and expertise of providers involved, if any). (Note: Only report in the abstract what the main paper is reporting. If this information is missing from the main body of text, consider adding it)

	1	2	3	4	5	
subitem not at all important	○	○	○	○	○	essential

Does your paper address subitem 1b-ii?

Copy and paste relevant sections from the manuscript abstract (include quotes in quotation marks "like this" to indicate direct quotes from your manuscript), or elaborate on this item by providing additional information not in the ms, or briefly explain why the item is not applicable/relevant for your study

"automated web-based randomized controlled trial"

1b-iii) Open vs. closed, web-based (self-assessment) vs. face-to-face assessments in the METHODS section of the ABSTRACT

Mention how participants were recruited (online vs. offline), e.g., from an open access [illegible]site or from a clinic or a closed online user group (closed usergroup trial), and clarify if [illegible] was a purely web-based trial, or there were face-to-face components (as part of the [illegible]vention or for assessment). Clearly say if outcomes were self-assessed through [illegible]stionnaires (as common in web-based trials). Note: In traditional offline trials, an open [illegible] (open-label trial) is a type of clinical trial in which both the researchers and [illegible]icipants know which treatment is being administered. To avoid confusion, use [illegible]nded” or “unblinded” to indicated the level of blinding instead of “open”, as “open” in web-based trials usually refers to “open access” (i.e. participants can self-enrol). (Note: Only report in the abstract what the main paper is reporting. If this information is missing from the main body of text, consider adding it)

scite_
1459
4
1011
1

	1	2	3	4	5	
subitem not at all important	○	○	○	○	○	essential

Does your paper address subitem 1b-iii?

Copy and paste relevant sections from the manuscript abstract (include quotes in quotation marks "like this" to indicate direct quotes from your manuscript), or elaborate on this item by providing additional information not in the ms, or briefly explain why the item is not applicable/relevant for your study

"A fully automated web-based randomized controlled trial."

1b-iv) RESULTS section in abstract must contain use data

Report number of participants enrolled/assessed in each group, the use/uptake of the intervention (e.g., attrition/adherence metrics, use over time, number of logins etc.), in addition to primary/secondary outcomes. (Note: Only report in the abstract what the main paper is reporting. If this information is missing from the main body of text, consider adding it)

	1	2	3	4	5	
subitem not at all important	○	○	○	○	○	essential

Does your paper address subitem 1b-iv?

Copy and paste relevant sections from the manuscript abstract (include quotes in quotation marks "like this" to indicate direct quotes from your manuscript), or elaborate on [illegible] item by providing additional information not in the ms, or briefly explain why the item is [illegible] applicable/relevant for your study

[illegible]v antwoord

scite_
1459
4
1011
1

1b-v) CONCLUSIONS/DISCUSSION in abstract for negative trials

Conclusions/Discussions in abstract for negative trials: Discuss the primary outcome - if the trial is negative (primary outcome not changed), and the intervention was not used, discuss whether negative results are attributable to lack of uptake and discuss reasons. (Note: Only report in the abstract what the main paper is reporting. If this information is missing from the main body of text, consider adding it)

	1	2	3	4	5	
subitem not at all important	○	○	○	○	○	essential

Does your paper address subitem 1b-v?

Copy and paste relevant sections from the manuscript abstract (include quotes in quotation marks "like this" to indicate direct quotes from your manuscript), or elaborate on this item by providing additional information not in the ms, or briefly explain why the item is not applicable/relevant for your study

Jouw antwoord

INTRODUCTION

scite_
1459
4
1011
1

In INTRODUCTION: Scientific background and explanation of rationale

) Problem and the type of system/solution

Describe the problem and the type of system/solution that is object of the study: intended as stand-alone intervention vs. incorporated in broader health care program? Intended for a particular patient population? Goals of the intervention, e.g., being more cost-effective to other interventions, replace or complement other solutions? (Note: Details about the intervention are provided in “Methods” under 5)

	1	2	3	4	5	
subitem not at all important	○	○	○	○	○	essential

Does your paper address subitem 2a-i? *

Copy and paste relevant sections from the manuscript (include quotes in quotation marks "like this" to indicate direct quotes from your manuscript), or elaborate on this item by providing additional information not in the ms, or briefly explain why the item is not applicable/relevant for your study

"Even though the majority (70%) of test-takers score higher than the threshold for severe suicidal ideation (SIDAS ≥ 21), very few of them (less than 10%) continue to the webpage about contacting the helpline by phone or chat. Due to the anonymous nature of the helpline's services, it is not possible to determine to what extent test-takers follow the advice about contacting the helpline."

"This study is focused on suicide prevention in a web-based environment. In this setting, persuasive eHealth technologies can be utilized to help and motivate people to reach out for help."

"With this study, we intended to test whether it is possible to increase service utilization among individuals with severe suicidal ideation by providing more tailored information."

2a-ii) Scientific background, rationale: What is known about the (type of) system

Scientific background, rationale: What is known about the (type of) system that is the object of the study (be sure to discuss the use of similar systems for other conditions/diagnoses, if appropiate), motivation for the study, i.e. what are the reasons for and what is the context for this specific study, from which stakeholder viewpoint is the study performed, potential impact of findings [2]. Briefly justify the choice of the comparator.

	1	2	3	4	5	
item not at all important	○	○	○	○	○	essential

scite_
1459
4
1011
1

Does your paper address subitem 2a-ii? *

Copy and paste relevant sections from the manuscript (include quotes in quotation marks "like this" to indicate direct quotes from your manuscript), or elaborate on this item by providing additional information not in the ms, or briefly explain why the item is not applicable/relevant for your study

"These persuasive systems can be defined as “computerized software or information systems designed to reinforce, change or shape attitudes or behaviors or both without using coercion or deception” [18]. Little research has been done on stimulating help-seeking behavior among anonymous and high-risk internet users. Therefore, motivating reluctant high-risk individuals in an online environment towards professional help is still relatively uncharted territory. "

"Our study is inspired by the work of Jaroszewski et al., [19] in which they evaluated a brief automated Barrier Reduction Intervention (BRI) designed to increase the use of crisis service referrals provided within the mental health app Koko. To the best of our knowledge, this study is the first automatic web-based RCT among people with severe suicidal ideation aimed to reduce barriers to a suicide prevention helpline."

2b) In INTRODUCTION: Specific objectives or hypotheses

Does your paper address CONSORT subitem 2b? *

Copy and paste relevant sections from the manuscript (include quotes in quotation marks "like this" to indicate direct quotes from your manuscript), or elaborate on this item by [illegible]iding additional information not in the ms, or briefly explain why the item is not [illegible]icable/relevant for your study

scite_
1459
4
1011
1

[illegible] aim of our study is two-fold: (1) to measure the effectiveness of a brief barrier [illegible]ction intervention (BRI) provided in the self-test motivating people with severe suicidal [illegible]ghts to contact the Dutch suicide prevention helpline and (2) to specifically evaluate the effectiveness of the BRI in increasing helpline use by high-risk groups for suicide such as men and middle-aged people (40-70 years)."

METHODS

3a) Description of trial design (such as parallel, factorial) including allocation ratio

Does your paper address CONSORT subitem 3a? *

Copy and paste relevant sections from the manuscript (include quotes in quotation marks "like this" to indicate direct quotes from your manuscript), or elaborate on this item by providing additional information not in the ms, or briefly explain why the item is not applicable/relevant for your study

"This study was designed as an automated web-based two-arm randomized controlled trial."

3b) Important changes to methods after trial commencement (such as eligibility criteria), with reasons

Does your paper address CONSORT subitem 3b? *

Copy and paste relevant sections from the manuscript (include quotes in quotation marks "like this" to indicate direct quotes from your manuscript), or elaborate on this item by providing additional information not in the ms, or briefly explain why the item is not applicable/relevant for your study

This is described in the Study Protocol (doi: 10.2196/41078).

3b-i) Bug fixes, Downtimes, Content Changes

[illegible] fixes, Downtimes, Content Changes: ehealth systems are often dynamic systems. A [illegible]cription of changes to methods therefore also includes important changes made on [illegible]ntervention or comparator during the trial (e.g., major bug fixes or changes in the [illegible]tionality or content) (5-iii) and other “unexpected events” that may have influenced [illegible]y design such as staff changes, system failures/downtimes, etc. [2].

scite_
1459
4
1011
1

	1	2	3	4	5	
subitem not at all important	○	○	○	○	○	essential

Does your paper address subitem 3b-i?

Copy and paste relevant sections from the manuscript (include quotes in quotation marks "like this" to indicate direct quotes from your manuscript), or elaborate on this item by providing additional information not in the ms, or briefly explain why the item is not applicable/relevant for your study

Jouw antwoord

4a) Eligibility criteria for participants

Does your paper address CONSORT subitem 4a? *

Copy and paste relevant sections from the manuscript (include quotes in quotation marks "like this" to indicate direct quotes from your manuscript), or elaborate on this item by providing additional information not in the ms, or briefly explain why the item is not applicable/relevant for your study

"Participants were excluded from the study if: they were younger than 16 years old, scored below the cut-off point for severe suicidal thoughts (SIDAS score < 21), or scored above the cut-off point for severe suicidal thoughts (SIDAS score ≥ 21) and reported being likely to contact the suicide prevention helpline."

scite_
1459
4
1011
1

[illegible]) Computer / Internet literacy

[illegible]puter / Internet literacy is often an implicit “de facto” eligibility criterion - this should be [illegible]icitly clarified.

	1	2	3	4	5	
subitem not at all important	○	○	○	○	○	essential

Does your paper address subitem 4a-i?

Copy and paste relevant sections from the manuscript (include quotes in quotation marks "like this" to indicate direct quotes from your manuscript), or elaborate on this item by providing additional information not in the ms, or briefly explain why the item is not applicable/relevant for your study

Jouw antwoord

4a-ii) Open vs. closed, web-based vs. face-to-face assessments:

Open vs. closed, web-based vs. face-to-face assessments: Mention how participants were recruited (online vs. offline), e.g., from an open access website or from a clinic, and clarify if this was a purely web-based trial, or there were face-to-face components (as part of the intervention or for assessment), i.e., to what degree got the study team to know the participant. In online-only trials, clarify if participants were quasi-anonymous and whether having multiple identities was possible or whether technical or logistical measures (e.g., cookies, email confirmation, phone calls) were used to detect/prevent these.

	1	2	3	4	5	
subitem not at all important	○	○	○	○	○	essential

Does your paper address subitem 4a-ii? *

scite_
1459
4
1011
1

[illegible]y and paste relevant sections from the manuscript (include quotes in quotation marks [illegible] this" to indicate direct quotes from your manuscript), or elaborate on this item by [illegible]iding additional information not in the ms, or briefly explain why the item is not [illegible]icable/relevant for your study

[illegible]nymous visitors of the self-test for suicidal thoughts on the website of the Dutch national suicide prevention helpline were asked if they wanted to help improve the current self-test by contributing to this study. Visitors of the website could select “Yes, I will participate in the study” or “No, I just want to fill in the self-test”. People who were willing to contribute to our study were redirected to an information page and were asked for their informed consent. Those who were not willing to participate were guided to the already existing self-test on the website."

4a-iii) Information giving during recruitment

Information given during recruitment. Specify how participants were briefed for recruitment and in the informed consent procedures (e.g., publish the informed consent documentation as appendix, see also item X26), as this information may have an effect on user self-selection, user expectation and may also bias results.

	1	2	3	4	5	
subitem not at all important	○	○	○	○	○	essential

Does your paper address subitem 4a-iii?

Copy and paste relevant sections from the manuscript (include quotes in quotation marks "like this" to indicate direct quotes from your manuscript), or elaborate on this item by providing additional information not in the ms, or briefly explain why the item is not applicable/relevant for your study

Jouw antwoord

4b) Settings and locations where the data were collected

scite_
1459
4
1011
1

[illegible]s your paper address CONSORT subitem 4b? *

[illegible] and paste relevant sections from the manuscript (include quotes in quotation marks [illegible] this" to indicate direct quotes from your manuscript), or elaborate on this item by [illegible]iding additional information not in the ms, or briefly explain why the item is not [illegible]icable/relevant for your study

The following terms are used in the manuscript: "self-test" "web-based"

4b-i) Report if outcomes were (self-)assessed through online questionnaires

Clearly report if outcomes were (self-)assessed through online questionnaires (as common in web-based trials) or otherwise.

	1	2	3	4	5	
subitem not at all important	○	○	○	○	○	essential

Does your paper address subitem 4b-i? *

Copy and paste relevant sections from the manuscript (include quotes in quotation marks "like this" to indicate direct quotes from your manuscript), or elaborate on this item by providing additional information not in the ms, or briefly explain why the item is not applicable/relevant for your study

"self-test" "Both respondents in the intervention and control condition were asked the following question"

4b-ii) Report how institutional affiliations are displayed

Report how institutional affiliations are displayed to potential participants [on ehealth media], as affiliations with prestigious hospitals or universities may affect volunteer rates, use, and reactions with regards to an intervention.(Not a required item – describe only if this may bias results)

scite_
1459
4
1011
1

	1	2	3	4	5	
item not at all important	○	○	○	○	○	essential

Does your paper address subitem 4b-ii?

Copy and paste relevant sections from the manuscript (include quotes in quotation marks "like this" to indicate direct quotes from your manuscript), or elaborate on this item by providing additional information not in the ms, or briefly explain why the item is not applicable/relevant for your study

Jouw antwoord

5) The interventions for each group with sufficient details to allow replication, including how and when they were actually administered

5-i) Mention names, credential, affiliations of the developers, sponsors, and owners

Mention names, credential, affiliations of the developers, sponsors, and owners [6] (if authors/evaluators are owners or developer of the software, this needs to be declared in a "Conflict of interest" section or mentioned elsewhere in the manuscript).

	1	2	3	4	5	
subitem not at all important	○	○	○	○	○	essential

Does your paper address subitem 5-i?

Copy and paste relevant sections from the manuscript (include quotes in quotation marks "like this" to indicate direct quotes from your manuscript), or elaborate on this item by providing additional information not in the ms, or briefly explain why the item is not applicable/relevant for your study

Jouw antwoord

scite_
1459
4
1011
1

[illegible] Describe the history/development process

[illegible]cribe the history/development process of the application and previous formative [illegible]uations (e.g., focus groups, usability testing), as these will have an impact on [illegible]ption/use rates and help with interpreting results.

	1	2	3	4	5	
subitem not at all important	○	○	○	○	○	essential

Does your paper address subitem 5-ii?

Copy and paste relevant sections from the manuscript (include quotes in quotation marks "like this" to indicate direct quotes from your manuscript), or elaborate on this item by providing additional information not in the ms, or briefly explain why the item is not applicable/relevant for your study

Jouw antwoord

5-iii) Revisions and updating

Revisions and updating. Clearly mention the date and/or version number of the application/intervention (and comparator, if applicable) evaluated, or describe whether the intervention underwent major changes during the evaluation process, or whether the development and/or content was “frozen” during the trial. Describe dynamic components such as news feeds or changing content which may have an impact on the replicability of the intervention (for unexpected events see item 3b).

	1	2	3	4	5	
subitem not at all important	○	○	○	○	○	essential

Does your paper address subitem 5-iii?

Copy and paste relevant sections from the manuscript (include quotes in quotation marks [illegible] this" to indicate direct quotes from your manuscript), or elaborate on this item by [illegible]iding additional information not in the ms, or briefly explain why the item is not [illegible]icable/relevant for your study

scite_
1459
4
1011
1

[illegible]v antwoord

5-iv) Quality assurance methods

Provide information on quality assurance methods to ensure accuracy and quality of information provided [1], if applicable.

	1	2	3	4	5	
subitem not at all important	○	○	○	○	○	essential

Does your paper address subitem 5-iv?

Copy and paste relevant sections from the manuscript (include quotes in quotation marks "like this" to indicate direct quotes from your manuscript), or elaborate on this item by providing additional information not in the ms, or briefly explain why the item is not applicable/relevant for your study

Jouw antwoord

5-v) Ensure replicability by publishing the source code, and/or providing screenshots/screen-capture video, and/or providing flowcharts of the algorithms used

Ensure replicability by publishing the source code, and/or providing screenshots/screen-capture video, and/or providing flowcharts of the algorithms used. Replicability (i.e., other researchers should in principle be able to replicate the study) is a hallmark of scientific reporting.

	1	2	3	4	5	
subitem not at all important	○	○	○	○	○	essential

Does your paper address subitem 5-v?

Copy and paste relevant sections from the manuscript (include quotes in quotation marks [illegible] this" to indicate direct quotes from your manuscript), or elaborate on this item by [illegible]iding additional information not in the ms, or briefly explain why the item is not [illegible]icable/relevant for your study

[illegible]v antwoord

scite_
1459
4
1011
1

5-vi) Digital preservation

Digital preservation: Provide the URL of the application, but as the intervention is likely to change or disappear over the course of the years; also make sure the intervention is archived (Internet Archive, webcitation.org, and/or publishing the source code or screenshots/videos alongside the article). As pages behind login screens cannot be archived, consider creating demo pages which are accessible without login.

	1	2	3	4	5	
subitem not at all important	○	○	○	○	○	essential

Does your paper address subitem 5-vi?

Copy and paste relevant sections from the manuscript (include quotes in quotation marks "like this" to indicate direct quotes from your manuscript), or elaborate on this item by providing additional information not in the ms, or briefly explain why the item is not applicable/relevant for your study

Jouw antwoord

5-vii) Access

Access: Describe how participants accessed the application, in what setting/context, if they had to pay (or were paid) or not, whether they had to be a member of specific group. If [illegible]wn, describe how participants obtained “access to the platform and Internet” [1]. To [illegible]ure access for editors/reviewers/readers, consider to provide a “backdoor” login [illegible]ount or demo mode for reviewers/readers to explore the application (also important for [illegible]iving purposes, see vi).

scite_
1459
4
1011
1

	1	2	3	4	5	
subitem not at all important	○	○	○	○	○	essential

Does your paper address subitem 5-vii? *

Copy and paste relevant sections from the manuscript (include quotes in quotation marks "like this" to indicate direct quotes from your manuscript), or elaborate on this item by providing additional information not in the ms, or briefly explain why the item is not applicable/relevant for your study

"Participants were recruited between October 7th and December 5th 2022. Anonymous visitors of the self-test for suicidal thoughts on the website of the Dutch national suicide prevention helpline were asked if they wanted to help improve the current self-test by contributing to this study."

5-viii) Mode of delivery, features/functionalities/components of the intervention and comparator, and the theoretical framework

Describe mode of delivery, features/functionalities/components of the intervention and comparator, and the theoretical framework [6] used to design them (instructional strategy [1], behaviour change techniques, persuasive features, etc., see e.g., [7, 8] for terminology). This includes an in-depth description of the content (including where it is coming from and who developed it) [1]," whether [and how] it is tailored to individual circumstances and allows users to track their progress and receive feedback" [6]. This also includes a description of communication delivery channels and – if computer-mediated communication is a component – whether communication was synchronous or asynchronous [6]. It also includes information on presentation strategies [1], including page design principles, average amount of text on pages, presence of hyperlinks to other resources, etc. [1].

	1	2	3	4	5	
item not at all important	○	○	○	○	○	essential

scite_
1459
4
1011
1

Does your paper address subitem 5-viii? *

Copy and paste relevant sections from the manuscript (include quotes in quotation marks "like this" to indicate direct quotes from your manuscript), or elaborate on this item by providing additional information not in the ms, or briefly explain why the item is not applicable/relevant for your study

This is described in the Study Protocol (doi: 10.2196/41078).

5-ix) Describe use parameters

Describe use parameters (e.g., intended “doses” and optimal timing for use). Clarify what instructions or recommendations were given to the user, e.g., regarding timing, frequency, heaviness of use, if any, or was the intervention used ad libitum.

	1	2	3	4	5	
subitem not at all important	○	○	○	○	○	essential

Does your paper address subitem 5-ix?

Copy and paste relevant sections from the manuscript (include quotes in quotation marks "like this" to indicate direct quotes from your manuscript), or elaborate on this item by providing additional information not in the ms, or briefly explain why the item is not applicable/relevant for your study

This is not applicable for this intervention as it was a brief one-time intervention.

5-x) Clarify the level of human involvement

Clarify the level of human involvement (care providers or health professionals, also technical assistance) in the e-intervention or as co-intervention (detail number and expertise of professionals involved, if any, as well as “type of assistance offered, the timing and frequency of the support, how it is initiated, and the medium by which the assistance [illegible]livered”. It may be necessary to distinguish between the level of human involvement [illegible]ired for the trial, and the level of human involvement required for a routine application [illegible]ide of a RCT setting (discuss under item 21 – generalizability).

scite_
1459
4
1011
1

	1	2	3	4	5	
subitem not at all important	○	○	○	○	○	essential

Does your paper address subitem 5-x?

Copy and paste relevant sections from the manuscript (include quotes in quotation marks "like this" to indicate direct quotes from your manuscript), or elaborate on this item by providing additional information not in the ms, or briefly explain why the item is not applicable/relevant for your study

Jouw antwoord

5-xi) Report any prompts/reminders used

Report any prompts/reminders used: Clarify if there were prompts (letters, emails, phone calls, SMS) to use the application, what triggered them, frequency etc. It may be necessary to distinguish between the level of prompts/reminders required for the trial, and the level of prompts/reminders for a routine application outside of a RCT setting (discuss under item 21 – generalizability).

	1	2	3	4	5	
subitem not at all important	○	○	○	○	○	essential

Does your paper address subitem 5-xi? *

Copy and paste relevant sections from the manuscript (include quotes in quotation marks "like this" to indicate direct quotes from your manuscript), or elaborate on this item by [illegible]iding additional information not in the ms, or briefly explain why the item is not [illegible]icable/relevant for your study

scite_
1459
4
1011
1

[illegible] is not applicable for this intervention as it was a brief one-time intervention.

5-xii) Describe any co-interventions (incl. training/support)

Describe any co-interventions (incl. training/support): Clearly state any interventions that are provided in addition to the targeted eHealth intervention, as ehealth intervention may not be designed as stand-alone intervention. This includes training sessions and support [1]. It may be necessary to distinguish between the level of training required for the trial, and the level of training for a routine application outside of a RCT setting (discuss under item 21 – generalizability.

	1	2	3	4	5	
subitem not at all important	○	○	○	○	○	essential

Does your paper address subitem 5-xii? *

Copy and paste relevant sections from the manuscript (include quotes in quotation marks "like this" to indicate direct quotes from your manuscript), or elaborate on this item by providing additional information not in the ms, or briefly explain why the item is not applicable/relevant for your study

This is not applicable for this intervention as it was a brief one-time intervention.

6a) Completely defined pre-specified primary and secondary outcome measures, including how and when they were assessed

scite_
1459
4
1011
1

[illegible]s your paper address CONSORT subitem 6a? *

[illegible]y and paste relevant sections from the manuscript (include quotes in quotation marks [illegible] this" to indicate direct quotes from your manuscript), or elaborate on this item by providing additional information not in the ms, or briefly explain why the item is not applicable/relevant for your study

This is in more detail described in the Study Protocol (doi: 10.2196/41078). The primary outcome measure was the use of a direct link to contact the helpline after completing the intervention or control condition (behavioral measure). Secondary outcomes were the self-reported likelihood of contacting the helpline (as a proxy for the behavioral measure) and satisfaction with the received self-test (using the Client Satisfaction Questionnaire CSQ-3).

6a-i) Online questionnaires: describe if they were validated for online use and apply CHERRIES items to describe how the questionnaires were designed/deployed

If outcomes were obtained through online questionnaires, describe if they were validated for online use and apply CHERRIES items to describe how the questionnaires were designed/deployed [9].

	1	2	3	4	5	
subitem not at all important	○	○	○	○	○	essential

Does your paper address subitem 6a-i?

Copy and paste relevant sections from manuscript text

Jouw antwoord

6a-ii) Describe whether and how "use" (including intensity of use/dosage) was defined/measured/monitored

Describe whether and how "use" (including intensity of use/dosage) was defined/measured/monitored (logins, logfile analysis, etc.). Use/adoption metrics are important process outcomes that should be reported in any ehealth trial.

	1	2	3	4	5	
item not at all important	○	○	○	○	○	essential

scite_
1459
4
1011
1

Does your paper address subitem 6a-ii?

Copy and paste relevant sections from manuscript text

Jouw antwoord

6a-iii) Describe whether, how, and when qualitative feedback from participants was obtained

Describe whether, how, and when qualitative feedback from participants was obtained (e.g., through emails, feedback forms, interviews, focus groups).

	1	2	3	4	5	
subitem not at all important	○	○	○	○	○	essential

Does your paper address subitem 6a-iii?

Copy and paste relevant sections from manuscript text

Jouw antwoord

6b) Any changes to trial outcomes after the trial commenced, with reasons

Does your paper address CONSORT subitem 6b? *

Copy and paste relevant sections from the manuscript (include quotes in quotation marks "like this" to indicate direct quotes from your manuscript), or elaborate on this item by providing additional information not in the ms, or briefly explain why the item is not [illegible]icable/relevant for your study

[illegible] is not applicable for this intervention as it was a brief one-time intervention.

scite_
1459
4
1011
1

7a) How sample size was determined

NPT: When applicable, details of whether and how the clustering by care provides or centers was addressed

7a-i) Describe whether and how expected attrition was taken into account when calculating the sample size

Describe whether and how expected attrition was taken into account when calculating the sample size.

	1	2	3	4	5	
subitem not at all important	○	○	○	○	○	essential

Does your paper address subitem 7a-i?

Copy and paste relevant sections from manuscript title (include quotes in quotation marks "like this" to indicate direct quotes from your manuscript), or elaborate on this item by providing additional information not in the ms, or briefly explain why the item is not applicable/relevant for your study

"The study protocol specified a sample size of 775 participants. We estimated that approximately 10% of participants would drop out during the intervention. We, therefore, expected that we would need to include at least 853 participants. Furthermore, we anticipated that approximately 30% of test-takers would score below the cut-off point for high risk of suicidal behavior and that 20% of test-takers would indicate a high probability of contacting the helpline in the entry screening questionnaire. Factoring in these projections, we determined that a recruitment goal of at least 1,706 respondents was necessary."

scite_
1459
4
1011
1

[illegible] When applicable, explanation of any interim analyses and stopping guidelines

[illegible]s your paper address CONSORT subitem 7b? *

Copy and paste relevant sections from the manuscript (include quotes in quotation marks "like this" to indicate direct quotes from your manuscript), or elaborate on this item by providing additional information not in the ms, or briefly explain why the item is not applicable/relevant for your study

This is not applicable for this intervention as it was a brief one-time intervention.

8a) Method used to generate the random allocation sequence

NPT: When applicable, how care providers were allocated to each trial group

Does your paper address CONSORT subitem 8a? *

Copy and paste relevant sections from the manuscript (include quotes in quotation marks "like this" to indicate direct quotes from your manuscript), or elaborate on this item by providing additional information not in the ms, or briefly explain why the item is not applicable/relevant for your study

"This study was designed as an automated web-based two-arm randomized controlled trial. Respondents with severe suicidal thoughts and no interest in contacting the helpline by phone or chat were randomly assigned to either a brief barrier reduction intervention (BRI) or received a general advisory text (care as usual)."

8b) Type of randomisation; details of any restriction (such as blocking and block size)

Does your paper address CONSORT subitem 8b? *

Copy and paste relevant sections from the manuscript (include quotes in quotation marks "like this" to indicate direct quotes from your manuscript), or elaborate on this item by providing additional information not in the ms, or briefly explain why the item is not applicable/relevant for your study

"This study was designed as an automated web-based two-arm randomized controlled trial. Respondents with severe suicidal thoughts and no interest in contacting the helpline by phone or chat were randomly assigned to either a brief barrier reduction intervention (BRI) or [illegible]ived a general advisory text (care as usual)."

scite_
1459
4
1011
1

[illegible]lechanism used to implement the random allocation sequence (such as [illegible]uentially numbered containers), describing any steps taken to conceal the sequence until interventions were assigned

Does your paper address CONSORT subitem 9? *

Copy and paste relevant sections from the manuscript (include quotes in quotation marks "like this" to indicate direct quotes from your manuscript), or elaborate on this item by providing additional information not in the ms, or briefly explain why the item is not applicable/relevant for your study

Automated web-based randomization.

10) Who generated the random allocation sequence, who enrolled participants, and who assigned participants to interventions

Does your paper address CONSORT subitem 10? *

Copy and paste relevant sections from the manuscript (include quotes in quotation marks "like this" to indicate direct quotes from your manuscript), or elaborate on this item by providing additional information not in the ms, or briefly explain why the item is not applicable/relevant for your study

Automated web-based randomization.

11a) If done, who was blinded after assignment to interventions (for example, participants, care providers, those assessing outcomes) and how

NPT: Whether or not administering co-interventions were blinded to group assignment

11a-i) Specify who was blinded, and who wasn't

Specify who was blinded, and who wasn't. Usually, in web-based trials it is not possible to blind the participants [1, 3] (this should be clearly acknowledged), but it may be possible to blind outcome assessors, those doing data analysis or those administering co-interventions (if any).

	1	2	3	4	5	
item not at all important	○	○	○	○	○	essential

scite_
1459
4
1011
1

Does your paper address subitem 11a-i? *

Copy and paste relevant sections from the manuscript (include quotes in quotation marks "like this" to indicate direct quotes from your manuscript), or elaborate on this item by providing additional information not in the ms, or briefly explain why the item is not applicable/relevant for your study

Automated web-based randomization and anonymous participants.

11a-ii) Discuss e.g., whether participants knew which intervention was the “intervention of interest” and which one was the “comparator”

Informed consent procedures (4a-ii) can create biases and certain expectations - discuss e.g., whether participants knew which intervention was the “intervention of interest” and which one was the “comparator”.

	1	2	3	4	5	
subitem not at all important	○	○	○	○	○	essential

Does your paper address subitem 11a-ii?

Copy and paste relevant sections from the manuscript (include quotes in quotation marks "like this" to indicate direct quotes from your manuscript), or elaborate on this item by providing additional information not in the ms, or briefly explain why the item is not applicable/relevant for your study

Jouw antwoord

11b) If relevant, description of the similarity of interventions

(this item is usually not relevant for ehealth trials as it refers to similarity of a placebo or sham intervention to a active medication/intervention)

scite_
1459
4
1011
1

Does your paper address CONSORT subitem 11b? *

Copy and paste relevant sections from the manuscript (include quotes in quotation marks "like this" to indicate direct quotes from your manuscript), or elaborate on this item by providing additional information not in the ms, or briefly explain why the item is not applicable/relevant for your study

"After the barrier question, the control group received a general advisory text. The text was similar to the advisory text individuals receive when they fill in the current self-test on the website of the helpline."
"Respondents in the intervention group received tailored information based on the respondents' self-reported barrier to contacting the helpline. The tailored information aimed to address common concerns and misconceptions about the helpline. The tailored advisory text for each barrier was structured as follows: (i) a friendly and informal overall advice in the tone of voice of the helpline; (ii) an anonymous quote from a help-seeker about his or her experience with help-seeking; and (ii) two quotes from counselors with or without lived experience. To educate people that they are certainly not the only ones suffering from suicidal thoughts, each advisory text contains a figure showing the text '113 receives an average of 450 requests for help per day'. As per the helpline's communication policy, each advisory texts emphasize the importance of talking about suicidal thoughts and taking the first step toward help."

12a) Statistical methods used to compare groups for primary and secondary outcomes

NPT: When applicable, details of whether and how the clustering by care providers or centers was addressed

scite_
1459
4
1011
1

[illegible]s your paper address CONSORT subitem 12a? *

[illegible] and paste relevant sections from the manuscript (include quotes in quotation marks [illegible] this" to indicate direct quotes from your manuscript), or elaborate on this item by [illegible]iding additional information not in the ms, or briefly explain why the item is not applicable/relevant for your study

"The first step of the data analysis was focused on verifying the randomization process and inspecting dropouts. To determine whether the control group and the intervention group, as well as the completers and non-completers, were comparable on baseline factors (gender, age, SIDAS-score, and in treatment or not) independent t-tests and chi-square tests were used. To test the hypothesis that respondents who received the brief BRI, were more likely to use the direct link to contact the suicide prevention helpline at the end of the trial than respondents in the control condition, chi-square analysis as well as multiple logistic regression analyses were used. For the secondary and tertiary outcome measures multiple linear regression analyses were conducted."

12a-i) Imputation techniques to deal with attrition / missing values

Imputation techniques to deal with attrition / missing values: Not all participants will use the intervention/comparator as intended and attrition is typically high in ehealth trials. Specify how participants who did not use the application or dropped out from the trial were treated in the statistical analysis (a complete case analysis is strongly discouraged, and simple imputation techniques such as LOCF may also be problematic [4]).

	1	2	3	4	5	
subitem not at all important	○	○	○	○	○	essential

Does your paper address subitem 12a-i? *

Copy and paste relevant sections from the manuscript (include quotes in quotation marks "like this" to indicate direct quotes from your manuscript), or elaborate on this item by providing additional information not in the ms, or briefly explain why the item is not applicable/relevant for your study

"As a sensitivity test, the outcome measures were also analyzed using intention-to-treat analysis. All missing values were imputed using the R package MICE [23], generating 10 independent datasets based on a maximum of 10 iterations each. To allow maximum imputation accuracy, we applied the classification and regression trees (cart) method for all variables and did not exclude any variables from the model a priori. Having generated 10 imputed datasets, missing values in the original dataset were replaced by the most frequent imputed value for factors, and by the average imputed value for numeric variables."

scite_
1459
4
1011
1

[illegible]) Methods for additional analyses, such as subgroup analyses and adjusted [illegible]lyses

Does your paper address CONSORT subitem 12b? *

Copy and paste relevant sections from the manuscript (include quotes in quotation marks "like this" to indicate direct quotes from your manuscript), or elaborate on this item by providing additional information not in the ms, or briefly explain why the item is not applicable/relevant for your study

"To determine whether the control group and the intervention group, as well as the completers and non-completers, were comparable on baseline factors (gender, age, SIDAS-score, and in treatment or not) independent t-tests and chi-square tests were used. To test the hypothesis that respondents who received the brief BRI, were more likely to use the direct link to contact the suicide prevention helpline at the end of the trial than respondents in the control condition, chi-square analysis as well as multiple logistic regression analyses were used. For the secondary and tertiary outcome measures multiple linear regression analyses were conducted."

X26) REB/IRB Approval and Ethical Considerations [recommended as subheading under "Methods"] (not a CONSORT item)

X26-i) Comment on ethics committee approval

	1	2	3	4	5	
subitem not at all important	○	○	○	○	○	essential

scite_
1459
4
1011
1

[illegible]s your paper address subitem X26-i?

[illegible]y and paste relevant sections from the manuscript (include quotes in quotation marks [illegible] this" to indicate direct quotes from your manuscript), or elaborate on this item by providing additional information not in the ms, or briefly explain why the item is not applicable/relevant for your study

Jouw antwoord

x26-ii) Outline informed consent procedures

Outline informed consent procedures e.g., if consent was obtained offline or online (how? Checkbox, etc.?), and what information was provided (see 4a-ii). See [6] for some items to be included in informed consent documents.

	1	2	3	4	5	
subitem not at all important	○	○	○	○	○	essential

Does your paper address subitem X26-ii?

Copy and paste relevant sections from the manuscript (include quotes in quotation marks "like this" to indicate direct quotes from your manuscript), or elaborate on this item by providing additional information not in the ms, or briefly explain why the item is not applicable/relevant for your study

Jouw antwoord

X26-iii) Safety and security procedures

Safety and security procedures, incl. privacy considerations, and any steps taken to reduce the likelihood or detection of harm (e.g., education and training, availability of a hotline)

	1	2	3	4	5	
item not at all important	○	○	○	○	○	essential

scite_
1459
4
1011
1

Does your paper address subitem X26-iii?

Copy and paste relevant sections from the manuscript (include quotes in quotation marks "like this" to indicate direct quotes from your manuscript), or elaborate on this item by providing additional information not in the ms, or briefly explain why the item is not applicable/relevant for your study

Jouw antwoord

RESULTS

13a) For each group, the numbers of participants who were randomly assigned, received intended treatment, and were analysed for the primary outcome

NPT: The number of care providers or centers performing the intervention in each group and the number of patients treated by each care provider in each center

Does your paper address CONSORT subitem 13a? *

Copy and paste relevant sections from the manuscript (include quotes in quotation marks "like this" to indicate direct quotes from your manuscript), or elaborate on this item by providing additional information not in the ms, or briefly explain why the item is not applicable/relevant for your study

The manuscript includes a flowchart including the number of participants in each step of the RCT.

13b) For each group, losses and exclusions after randomisation, together with reasons

Does your paper address CONSORT subitem 13b? (NOTE: Preferably, this is shown in a CONSORT flow diagram) *

Copy and paste relevant sections from the manuscript (include quotes in quotation marks "like this" to indicate direct quotes from your manuscript), or elaborate on this item by [illegible]iding additional information not in the ms, or briefly explain why the item is not [illegible]icable/relevant for your study

[illegible]v antwoord

scite_
1459
4
1011
1

13b-i) Attrition diagram

Strongly recommended: An attrition diagram (e.g., proportion of participants still logging in or using the intervention/comparator in each group plotted over time, similar to a survival curve) or other figures or tables demonstrating usage/dose/engagement.

	1	2	3	4	5	
subitem not at all important	○	○	○	○	○	essential

Does your paper address subitem 13b-i?

Copy and paste relevant sections from the manuscript or cite the figure number if applicable (include quotes in quotation marks "like this" to indicate direct quotes from your manuscript), or elaborate on this item by providing additional information not in the ms, or briefly explain why the item is not applicable/relevant for your study

Jouw antwoord

14a) Dates defining the periods of recruitment and follow-up

Does your paper address CONSORT subitem 14a? *

Copy and paste relevant sections from the manuscript (include quotes in quotation marks "like this" to indicate direct quotes from your manuscript), or elaborate on this item by providing additional information not in the ms, or briefly explain why the item is not applicable/relevant for your study

"Participants were recruited between October 7th and December 5th 2022." Brief one-time intervention.

14a-i) Indicate if critical “secular events” fell into the study period

Indicate if critical “secular events” fell into the study period, e.g., significant changes in [illegible]net resources available or “changes in computer hardware or Internet delivery [illegible]urces”

scite_
1459
4
1011
1

	1	2	3	4	5	
subitem not at all important	○	○	○	○	○	essential

Does your paper address subitem 14a-i?

Copy and paste relevant sections from the manuscript (include quotes in quotation marks "like this" to indicate direct quotes from your manuscript), or elaborate on this item by providing additional information not in the ms, or briefly explain why the item is not applicable/relevant for your study

Jouw antwoord

14b) Why the trial ended or was stopped (early)

Does your paper address CONSORT subitem 14b? *

Copy and paste relevant sections from the manuscript (include quotes in quotation marks "like this" to indicate direct quotes from your manuscript), or elaborate on this item by providing additional information not in the ms, or briefly explain why the item is not applicable/relevant for your study

Not applicable, intervention did not stop earlier than planned.

15) A table showing baseline demographic and clinical characteristics for each group

NPT: When applicable, a description of care providers (case volume, qualification, expertise, etc.) and centers (volume) in each group

Does your paper address CONSORT subitem 15? *

Copy and paste relevant sections from the manuscript (include quotes in quotation marks "like this" to indicate direct quotes from your manuscript), or elaborate on this item by providing additional information not in the ms, or briefly explain why the item is not applicable/relevant for your study

[illegible]uscript includes table with respondents' characteristics (Table 1).

scite_
1459
4
1011
1

[illegible]) Report demographics associated with digital divide issues

[illegible] ehealth trials it is particularly important to report demographics associated with digital divide issues, such as age, education, gender, social-economic status, computer/Internet/ehealth literacy of the participants, if known.

	1	2	3	4	5	
subitem not at all important	○	○	○	○	○	essential

Does your paper address subitem 15-i? *

Copy and paste relevant sections from the manuscript (include quotes in quotation marks "like this" to indicate direct quotes from your manuscript), or elaborate on this item by providing additional information not in the ms, or briefly explain why the item is not applicable/relevant for your study

Manuscript includes table with respondents' characteristics (Table 1).

16) For each group, number of participants (denominator) included in each analysis and whether the analysis was by original assigned groups

16-i) Report multiple "denominators" and provide definitions

Report multiple "denominators" and provide definitions: Report N's (and effect sizes) "across a range of study participation [and use] thresholds" [1], e.g., N exposed, N consented, N used more than x times, N used more than y weeks, N participants "used" the intervention/comparator at specific pre-defined time points of interest (in absolute and relative numbers per group). Always clearly define "use" of the intervention.

	1	2	3	4	5	
subitem not at all important	○	○	○	○	○	essential

scite_
1459
4
1011
1

[illegible]s your paper address subitem 16-i? *

[illegible]y and paste relevant sections from the manuscript (include quotes in quotation marks [illegible] this" to indicate direct quotes from your manuscript), or elaborate on this item by [illegible]iding additional information not in the ms, or briefly explain why the item is not applicable/relevant for your study

Yes, each table and text describing analyses and results, includes the N.

16-ii) Primary analysis should be intent-to-treat

Primary analysis should be intent-to-treat, secondary analyses could include comparing only “users”, with the appropriate caveats that this is no longer a randomized sample (see 18-i).

	1	2	3	4	5	
subitem not at all important	○	○	○	○	○	essential

Does your paper address subitem 16-ii?

Copy and paste relevant sections from the manuscript (include quotes in quotation marks "like this" to indicate direct quotes from your manuscript), or elaborate on this item by providing additional information not in the ms, or briefly explain why the item is not applicable/relevant for your study

Jouw antwoord

17a) For each primary and secondary outcome, results for each group, and the estimated effect size and its precision (such as 95% confidence interval)

scite_
1459
4
1011
1

[illegible]s your paper address CONSORT subitem 17a? *

[illegible] and paste relevant sections from the manuscript (include quotes in quotation marks [illegible] this" to indicate direct quotes from your manuscript), or elaborate on this item by [illegible]iding additional information not in the ms, or briefly explain why the item is not [illegible]icable/relevant for your study

Yes, estimated effect sizes and its precision (by using the 95%CI) is reported.

17a-i) Presentation of process outcomes such as metrics of use and intensity of use

In addition to primary/secondary (clinical) outcomes, the presentation of process outcomes such as metrics of use and intensity of use (dose, exposure) and their operational definitions is critical. This does not only refer to metrics of attrition (13-b) (often a binary variable), but also to more continuous exposure metrics such as "average session length". These must be accompanied by a technical description how a metric like a "session" is defined (e.g., timeout after idle time) [1] (report under item 6a).

	1	2	3	4	5	
subitem not at all important	○	○	○	○	○	essential

Does your paper address subitem 17a-i?

Copy and paste relevant sections from the manuscript (include quotes in quotation marks "like this" to indicate direct quotes from your manuscript), or elaborate on this item by providing additional information not in the ms, or briefly explain why the item is not applicable/relevant for your study

Jouw antwoord

17b) For binary outcomes, presentation of both absolute and relative effect sizes is [illegible]ommended

scite_
1459
4
1011
1

[illegible]s your paper address CONSORT subitem 17b? *

[illegible]y and paste relevant sections from the manuscript (include quotes in quotation marks "like this" to indicate direct quotes from your manuscript), or elaborate on this item by providing additional information not in the ms, or briefly explain why the item is not applicable/relevant for your study

Yes, both absolute and relative effect sizes are reported.

18) Results of any other analyses performed, including subgroup analyses and adjusted analyses, distinguishing pre-specified from exploratory

Does your paper address CONSORT subitem 18? *

Copy and paste relevant sections from the manuscript (include quotes in quotation marks "like this" to indicate direct quotes from your manuscript), or elaborate on this item by providing additional information not in the ms, or briefly explain why the item is not applicable/relevant for your study

Manuscript includes both complete case analyses as well as Intention-to-treat analysis (ITT).

18-i) Subgroup analysis of comparing only users

A subgroup analysis of comparing only users is not uncommon in ehealth trials, but if done, it must be stressed that this is a self-selected sample and no longer an unbiased sample from a randomized trial (see 16-iii).

	1	2	3	4	5	
subitem not at all important	○	○	○	○	○	essential

Does your paper address subitem 18-i?

Copy and paste relevant sections from the manuscript (include quotes in quotation marks "like this" to indicate direct quotes from your manuscript), or elaborate on this item by providing additional information not in the ms, or briefly explain why the item is not [illegible]licable/relevant for your study

scite_
1459
4
1011
1

[illegible] antwoord

19) All important harms or unintended effects in each group

(for specific guidance see CONSORT for harms)

Does your paper address CONSORT subitem 19? *

Copy and paste relevant sections from the manuscript (include quotes in quotation marks "like this" to indicate direct quotes from your manuscript), or elaborate on this item by providing additional information not in the ms, or briefly explain why the item is not applicable/relevant for your study

Brief, one time intervention on website with anonymous respondents.

19-i) Include privacy breaches, technical problems

Include privacy breaches, technical problems. This does not only include physical “harm” to participants, but also incidents such as perceived or real privacy breaches [1], technical problems, and other unexpected/unintended incidents. “Unintended effects” also includes unintended positive effects [2].

	1	2	3	4	5	
subitem not at all important	○	○	○	○	○	essential

Does your paper address subitem 19-i?

Copy and paste relevant sections from the manuscript (include quotes in quotation marks "like this" to indicate direct quotes from your manuscript), or elaborate on this item by providing additional information not in the ms, or briefly explain why the item is not [illegible]icable/relevant for your study

[illegible]v antwoord

scite_
1459
4
1011
1

19-ii) Include qualitative feedback from participants or observations from staff/researchers

Include qualitative feedback from participants or observations from staff/researchers, if available, on strengths and shortcomings of the application, especially if they point to unintended/unexpected effects or uses. This includes (if available) reasons for why people did or did not use the application as intended by the developers.

	1	2	3	4	5	
subitem not at all important	○	○	○	○	○	essential

Does your paper address subitem 19-ii?

Copy and paste relevant sections from the manuscript (include quotes in quotation marks "like this" to indicate direct quotes from your manuscript), or elaborate on this item by providing additional information not in the ms, or briefly explain why the item is not applicable/relevant for your study

Jouw antwoord

DISCUSSION

22) Interpretation consistent with results, balancing benefits and harms, and considering other relevant evidence

NPT: In addition, take into account the choice of the comparator, lack of or partial blinding, and unequal expertise of care providers or centers in each group

22-i) Restate study questions and summarize the answers suggested by the data, starting with primary outcomes and process outcomes (use)

Restate study questions and summarize the answers suggested by the data, starting with primary outcomes and process outcomes (use).

	1	2	3	4	5	
item not at all important	○	○	○	○	○	essential

scite_
1459
4
1011
1

Does your paper address subitem 22-i? *

Copy and paste relevant sections from the manuscript (include quotes in quotation marks "like this" to indicate direct quotes from your manuscript), or elaborate on this item by providing additional information not in the ms, or briefly explain why the item is not applicable/relevant for your study

"The aim of our study was two-fold: (1) to measure the effectiveness of a brief barrier reduction intervention (BRI) provided in the self-test motivating people with severe suicidal thoughts to contact the Dutch suicide prevention helpline and (2) to specifically evaluate the effectiveness of the BRI in increasing service use by high-risk groups for suicide such as men and middle-aged people."
"Respondents' characteristics were in line with expectations based on previous research on the helpline. Namely, they were mainly female, young and having severe suicidal thoughts. [5–7]. Almost half of the respondents (46.4%, N = 358) were not receiving treatment from mental health services at the time. This percentage was higher among the male (60.7%, N = 148) than the female respondents (38.9%, N = 192). At the time of the study, 11.9% (N = 92) of the respondents was on a waiting list for mental health services (Men: 8.6%, N = 21; women: 13.8%, N = 68)."
"In this study, 2,124 website visitors completed the SIDAS and the demographic questions in the screening phase. More than three-quarters (78%) scored higher than the cut-off point for severe suicidal thoughts (SIDAS ≥ 21). The mean SIDAS score in the screening phase was 28.4. Among the eventual study sample, the mean SIDAS score was 33, as the BRI was intended for the high-risk group."
"The results indicated no difference in the use of a direct link to the helpline immediately at the end of the few-minute-long trial. However, the results do show an impact on one's self-reported likelihood of contacting the helpline. In addition, respondents who received the BRI were more positive about the self-test than those in the control condition. When looking at the high-risk groups men and middle-aged people specifically, the results were comparable to the whole group of respondents."

scite_
1459
4
1011
1

[illegible]i) Highlight unanswered new questions, suggest future research

Highlight unanswered new questions, suggest future research.

	1	2	3	4	5	
subitem not at all important	○	○	○	○	○	essential

Does your paper address subitem 22-ii?

Copy and paste relevant sections from the manuscript (include quotes in quotation marks "like this" to indicate direct quotes from your manuscript), or elaborate on this item by providing additional information not in the ms, or briefly explain why the item is not applicable/relevant for your study

Jouw antwoord

20) Trial limitations, addressing sources of potential bias, imprecision, and, if relevant, multiplicity of analyses

20-i) Typical limitations in ehealth trials

Typical limitations in ehealth trials: Participants in ehealth trials are rarely blinded. Ehealth trials often look at a multiplicity of outcomes, increasing risk for a Type I error. Discuss biases due to non-use of the intervention/usability issues, biases through informed consent procedures, unexpected events.

	1	2	3	4	5	
subitem not at all important	○	○	○	○	○	essential

scite_
1459
4
1011
1

[illegible]s your paper address subitem 20-i? *

[illegible]y and paste relevant sections from the manuscript (include quotes in quotation marks [illegible] this" to indicate direct quotes from your manuscript), or elaborate on this item by [illegible]iding additional information not in the ms, or briefly explain why the item is not [illegible]icable/relevant for your study

"Second, due to the anonymity of the suicide prevention helpline, it was not possible to include a follow-up measurement. It is therefore not feasible to determine if those who did not use the link to the helpline after the intervention, did contact the helpline at a later moment in time. Because of this, the self-reported likelihood of contacting the helpline has also been measured as well as the use of the direct link. Third, there were relatively high drop-out rates during the RCT (36.8%). This is not entirely unexpected for a short, anonymous online survey but still noteworthy. In order to assess the robustness of the results, analyses were carried out on the complete cases (per protocol) as well as on an imputed dataset (intention-to-treat)."

21) Generalisability (external validity, applicability) of the trial findings

NPT: External validity of the trial findings according to the intervention, comparators, patients, and care providers or centers involved in the trial

21-i) Generalizability to other populations

Generalizability to other populations: In particular, discuss generalizability to a general Internet population, outside of a RCT setting, and general patient population, including applicability of the study results for other organizations

	1	2	3	4	5	
subitem not at all important	○	○	○	○	○	essential

Does your paper address subitem 21-i?

Copy and paste relevant sections from the manuscript (include quotes in quotation marks "like this" to indicate direct quotes from your manuscript), or elaborate on this item by providing additional information not in the ms, or briefly explain why the item is not applicable/relevant for your study

Jouw antwoord

scite_
1459
4
1011
1

[illegible]i) Discuss if there were elements in the RCT that would be different in a routine [illegible]lication setting

[illegible]uss if there were elements in the RCT that would be different in a routine application [illegible]ng (e.g., prompts/reminders, more human involvement, training sessions or other co-[illegible]ventions) and what impact the omission of these elements could have on use, adoption, or outcomes if the intervention is applied outside of a RCT setting.

	1	2	3	4	5	
subitem not at all important	○	○	○	○	○	essential

Does your paper address subitem 21-ii?

Copy and paste relevant sections from the manuscript (include quotes in quotation marks "like this" to indicate direct quotes from your manuscript), or elaborate on this item by providing additional information not in the ms, or briefly explain why the item is not applicable/relevant for your study

Jouw antwoord

OTHER INFORMATION

23) Registration number and name of trial registry

Does your paper address CONSORT subitem 23? *

Copy and paste relevant sections from the manuscript (include quotes in quotation marks "like this" to indicate direct quotes from your manuscript), or elaborate on this item by providing additional information not in the ms, or briefly explain why the item is not applicable/relevant for your study

"This study was reviewed and approved by the Medical Ethics Committee of the Vrije Universiteit Medical Centre (registration number 2021.0443) and is registered at www.clinicaltrials.gov (NCT05458830)."

[illegible]istration: ClinicalTrials.gov NCT05458830,
[illegible]s://clinicaltrials.gov/study/NCT05458830
[illegible]national registered report identifier (irrid): PRR1-10.2196/41078."

scite_
1459
4
1011
1

24) Where the full trial protocol can be accessed, if available

Does your paper address CONSORT subitem 24? *

Cite a Multimedia Appendix, other reference, or copy and paste relevant sections from the manuscript (include quotes in quotation marks "like this" to indicate direct quotes from your manuscript), or elaborate on this item by providing additional information not in the ms, or briefly explain why the item is not applicable/relevant for your study

"JMIR Res Protoc 2023;12:e41078, doi: 10.2196/41078"

25) Sources of funding and other support (such as supply of drugs), role of funders

Does your paper address CONSORT subitem 25? *

Copy and paste relevant sections from the manuscript (include quotes in quotation marks "like this" to indicate direct quotes from your manuscript), or elaborate on this item by providing additional information not in the ms, or briefly explain why the item is not applicable/relevant for your study

"This research was funded by 113 Suicide Prevention which is in itself mainly funded by the Ministry of Health, Welfare & Sports of the Netherlands. All authors report no financial relationships with commercial interests."

X27) Conflicts of Interest (not a CONSORT item)

scite_
1459
4
1011
1

[illegible]-i) State the relation of the study team towards the system being evaluated

[illegible]ddition to the usual declaration of interests (financial or otherwise), also state the [illegible]ion of the study team towards the system being evaluated, i.e., state if the authors/evaluators are distinct from or identical with the developers/sponsors of the intervention.

	1	2	3	4	5	
subitem not at all important	○	○	○	○	○	essential

Does your paper address subitem X27-i?

Copy and paste relevant sections from the manuscript (include quotes in quotation marks "like this" to indicate direct quotes from your manuscript), or elaborate on this item by providing additional information not in the ms, or briefly explain why the item is not applicable/relevant for your study

Jouw antwoord

About the CONSORT EHEALTH checklist

As a result of using this checklist, did you make changes in your manuscript? *

- ○ yes, major changes
- ◉ yes, minor changes
- ○ no

What were the most important changes you made as a result of using this checklist?

[illegible]v antwoord

scite_
1459
4
1011
1

[illegible]v much time did you spend on going through the checklist INCLUDING making changes in your manuscript *

approximately 60 minutes.

As a result of using this checklist, do you think your manuscript has improved? *

- ◯ yes
- ◯ no
- ◉ Anders: based on checklist and comments from the editor.

Would you like to become involved in the CONSORT EHEALTH group?

This would involve for example becoming involved in participating in a workshop and writing an "Explanation and Elaboration" document

- ◯ yes
- ◯ no
- ◯ Anders:

Any other comments or questions on CONSORT EHEALTH

Jouw antwoord

scite_
1459
4
1011
1

[illegible]P - Save this form as PDF before you click submit

[illegible]enerate a record that you filled in this form, we recommend to generate a PDF of this [illegible] (on a Mac, simply select "print" and then select "print as PDF") before you submit it.

[illegible]en you submit your (revised) paper to JMIR, please upload the PDF as supplementary file.

Don't worry if some text in the textboxes is cut off, as we still have the complete information in our database. Thank you!

Final step: Click submit !

Click submit so we have your answers in our database!

Verzenden Formulier wissen

Verzend nooit wachtwoorden via Google Formulieren.

Deze content is niet gemaakt of goedgekeurd door Google. Misbruik rapporteren - Servicevoorwaarden - Privacybeleid

Google Formulieren

scite_
1459
4
1011
1

scite_
1459
4
1011
1
